# Clinical effectiveness of one ultrasound guided intra-articular corticosteroid and local anaesthetic injection in addition to advice and education for hip osteoarthritis (HIT trial): single blind, parallel group, three arm, randomised controlled trial

**DOI:** 10.1136/bmj-2021-068446

**Published:** 2022-04-06

**Authors:** Zoe Paskins, Kieran Bromley, Martyn Lewis, Gemma Hughes, Emily Hughes, Susie Hennings, Andrea Cherrington, Alison Hall, Melanie A Holden, Kay Stevenson, Ajit Menon, Philip Roberts, George Peat, Clare Jinks, Jesse Kigozi, Raymond Oppong, Nadine E Foster, Christian D Mallen, Edward Roddy

**Affiliations:** 1 Primary Care Centre Versus Arthritis, School of Medicine, Keele University, Keele, UK; 2 Keele Clinical Trials Unit, Keele University, Keele, UK; 3 Haywood Academic Rheumatology Centre, Midlands Partnership NHS Foundation Trust, Stoke-on-Trent, UK; 4 University Hospitals North Midlands, Stoke-on-Trent, UK; 5 Health Economics Unit, University of Birmingham, Birmingham, UK; 6 Surgical Treatment And Rehabilitation Service (STARS), Research and Education Alliance, University of Queensland and Metro North Hospital and Health Service, Brisbane QLD, Australia; Correspondence to: Z Paskins z.paskins@keele.ac.uk (or @zpaskins on Twitter)

## Abstract

**Objective:**

To compare the clinical effectiveness of adding a single ultrasound guided intra-articular hip injection of corticosteroid and local anaesthetic to advice and education in adults with hip osteoarthritis.

**Design:**

Pragmatic, three arm, parallel group, single blind, randomised controlled trial.

**Setting:**

Two community musculoskeletal services in England.

**Participants:**

199 adults aged ≥40 years with hip osteoarthritis and at least moderate pain: 67 were randomly assigned to receive advice and education (best current treatment (BCT)), 66 to BCT plus ultrasound guided injection of triamcinolone and lidocaine, and 66 to BCT plus ultrasound guided injection of lidocaine.

**Interventions:**

BCT alone, BCT plus ultrasound guided intra-articular hip injection of 40 mg triamcinolone acetonide and 4 mL 1% lidocaine hydrochloride, or BCT plus ultrasound guided intra-articular hip injection of 5 mL 1% lidocaine. Participants in the ultrasound guided arms were masked to the injection they received.

**Main outcome measures:**

The primary outcome was self-reported current intensity of hip pain (0-10 Numerical Rating Scale) over six months. Outcomes were self-reported at two weeks and at two, four, and six months.

**Results:**

Mean age of the study sample was 62.8 years (standard deviation 10.0) and 113 (57%) were women. Average weighted follow-up rate across time points was 93%. Greater mean improvement in hip pain intensity over six months was reported with BCT plus ultrasound-triamcinolone-lidocaine compared with BCT: mean difference −1.43 (95% confidence interval −2.15 to −0.72), P<0.001; standardised mean difference −0.55 (−0.82 to −0.27). No difference in hip pain intensity over six months was reported between BCT plus ultrasound-triamcinolone-lidocaine compared with BCT plus ultrasound-lidocaine (−0.52 (−1.21 to 0.18)). The presence of ultrasound confirmed synovitis or effusion was associated with a significant interaction effect favouring BCT plus ultrasound-triamcinolone-lidocaine (−1.70 (−3.10 to −0.30)). One participant in the BCT plus ultrasound-triamcinolone-lidocaine group with a bioprosthetic aortic valve died from subacute bacterial endocarditis four months after the intervention, deemed possibly related to the trial treatment.

**Conclusions:**

Ultrasound guided intra-articular hip injection of triamcinolone is a treatment option to add to BCT for people with hip osteoarthritis.

**Trial registration:**

EudraCT 2014-003412-37; ISRCTN50550256.

**Figure:**
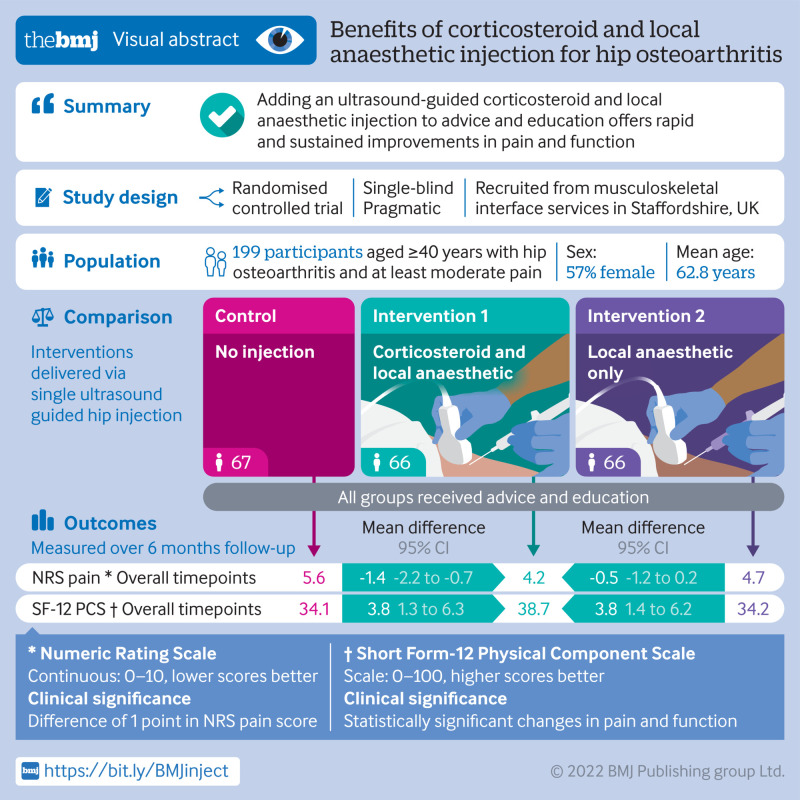


## Introduction

Hip osteoarthritis affects a substantial and growing number of people worldwide and is a leading cause of global disability,^1^ with a large proportion of those affected experiencing persistent pain, functional loss, and impaired quality of life.^2^ In 2019, more than 100 000 primary total hip replacements were undertaken in the UK, at a cost exceeding £500m (€596m; $659m).^3^ ^4^ Ninety per cent of these surgeries were for osteoarthritis. Although not all patients with hip osteoarthritis require surgery, the numbers of hip replacements continue to increase.^5^ Patients with hip osteoarthritis are typically treated in primary care for several years before surgical referral, with evidence suggesting that primary care management is suboptimal.^6^

Guidance from the National Institute for Health and Care Excellence advises combining non-drug and drug approaches, with education, exercise, and weight reduction as core treatments.^7^ Analgesic options include paracetamol (acetaminophen), with intra-articular corticosteroid injection recommended as an adjunct if pain is moderate to severe. However, guidelines highlight that the clinical and economic evidence supporting the use of intra-articular hip corticosteroid injection is limited and conflicting,^7^ and recommend intra-articular corticosteroid for knee osteoarthritis but not for hip osteoarthritis.^8^ A systematic review identified only five trials of corticosteroid injection in people with hip osteoarthritis, which were limited by recruitment from secondary care (eg, surgical waiting lists), and small sample sizes (n=36-101).^9^ Two randomised controlled trials showed clinical benefits of corticosteroid and local anaesthetic injection, compared with control injection, at eight weeks after injection,^10^ ^11^ with only one trial reporting outcomes beyond 8 weeks (non-significant reduction in pain between intervention and control).^12^ These previous randomised controlled trials compared corticosteroid injection with either local anaesthetic or saline but only one used the comparison of standard care.^9^ In addition to uncertainties about patient selection and effectiveness, use of intra-articular corticosteroid injection can also be limited by availability.^13^ Hip injections require imaging guidance, either using fluoroscopy or ultrasound, to improve the accuracy of placement. Ultrasonography avoids ionising radiation or a contrast agent and is more widely available than fluoroscopy because ultrasound guided injections at other sites are commonly performed in outpatient settings. However, only two randomised controlled trials of corticosteroid injections in hip osteoarthritis used ultrasound guidance.^10^ ^12^

In this study the clinical effectiveness of best current treatment (BCT) plus an ultrasound guided intra-articular hip injection of triamcinolone and lidocaine or of lidocaine alone was compared with BCT. 

## Methods

### Study design

The Hip Injection Trial (HIT), a pragmatic, three arm, parallel group, single blind, randomised controlled trial, was conducted within the National Health Service in two community based musculoskeletal services in England. The trial included a linked qualitative study and economic analysis (both reported separately). The protocol has been published previously.^14^

### Participants

We recruited participants after referral from primary care to orthopaedics, rheumatology, or two musculoskeletal interface services, as well as directly from general practice. We sent potentially eligible patients study information and invited them to attend hip clinics within the two musculoskeletal interface services. The patients were screened, and after consent had been obtained, they were treated by trained rheumatologists or extended scope physiotherapists.

Patients were eligible if they were aged ≥40 years with moderate to severe pain attributable to hip osteoarthritis present for six weeks or more and occurring on most days in the past month. Originally, we required participants to have a recorded hip pain intensity of ≥4 on a 0-10 Numerical Rating Scale on the day of assessment. In response to a suboptimal number of participants recruited (n=48), this eligibility criterion was amended on 7 November 2016, with external steering committee approval, to require an average pain Numerical Rating Scale score of ≥4 over the preceding two weeks, with a minimum of 1 on the day of assessment, to take into account day-to-day variability in osteoarthritis symptoms.^14^ We based a diagnosis of hip osteoarthritis on clinical history and examination, supported by radiographical evidence within the past two years. We asked patients with bilateral symptoms to select the most severely affected hip for treatment.

Exclusion criteria were hip pain from other causes (eg, trochanteric bursitis, osteonecrosis, or pain referred from back); substantial injury to the affected hip within the past three months; malignancy (as a possible cause of hip pain); inflammatory arthritis; corticosteroid injection into the affected hip or ipsilateral trochanteric bursa within three months; any condition requiring regular oral steroid use; anticoagulant treatment; previous surgery or infection of the affected hip; suspicion of current infection; allergy or contraindication to the study drugs; pregnancy; unwillingness to receive an injection; or inability to give full informed consent.^14^

### Randomisation and masking

We randomly assigned participants to one of three treatment arms (1:1:1): BCT, BCT plus ultrasound guided intra-articular hip injection of-triamcinolone and lidocaine, or BCT plus ultrasound guided intra-articular hip injection of-lidocaine, using random permuted blocks of three and six, via Keele Clinical Trial Unit’s secure web based randomisation service. Allocation concealment was ensured through remote computer generation of the randomisation sequence.

We did not mask participants and those who administered the injections to receipt of an injection, but we did mask the participants in the two injection groups and the assessors to the type of injection. Injections were prepared before participants entered the room. Participants were supine with the ultrasound monitor placed behind their head and hence could not view the injection site, syringes, or monitor. We masked statisticians and research nurses to the intervention allocation. To evaluate the success of blinding, we asked participants which injection they thought they had received immediately after the injection and recorded this information.

### Procedures

After consent had been obtained, all participants completed baseline data collection and received BCT at the same clinic visit, by the assessing clinician, before randomisation. BCT comprised written information (Versus Arthritis Osteoarthritis leaflet), a bespoke leaflet on exercise and functional activities,^15^ and personalised advice and information about weight loss, exercise, footwear, walking aids, and pain management.

A clinical administrator then randomly assigned participants to an intervention. People who were randomly assigned to BCT received no further treatment. Participants randomly assigned to BCT plus ultrasound-triamcinolone-lidocaine received one ultrasound guided injection of triamcinolone acetonide 40 mg/mL sterile, aqueous solution (Bristol Myers Squibb, Dublin, Ireland) and 4 mL 1% lidocaine hydrochloride (Hameln Pharma, Gloucester, UK), and people who were randomly assigned to BCT plus ultrasound-lidocaine received one ultrasound-guided injection of 5 mL 1% lidocaine hydrochloride (Hameln Pharma, Gloucester, UK); the same number of syringes were used in both injection groups to maintain blinding. Within the same clinic visit as the assessment and BCT, a different clinician (trained rheumatologist, extended scope physiotherapist, or sonographer) delivered the injections using an aseptic technique. Participants lay supine with legs extended into a neutral position. The skin was cleaned with chlorhexidine 0.5% solution. The transducer was covered with gel and a sterile sheath. Bilateral ultrasound images were obtained and scored for the presence of synovitis and effusion. The clinician located the anterior capsule of the hip joint using ultrasound and introduced 3 mL of 1% lidocaine to the overlying skin and superficial soft tissues using a 25G needle. A 22G spinal needle was inserted, under ultrasound guidance, until its tip entered the anterior joint capsule. The clinician injected 1 mL of 1% lidocaine into the hip to confirm correct placement, followed by either 40 mg triamcinolone (1 mL volume) with a further 3 mL of 1% lidocaine, or 4 mL of 1% lidocaine, both showing capsular distension by the fluid under ultrasound (total intracapsular volume 5 mL).^14^ Any other non-injection treatment (eg, analgesia, physiotherapy, or orthopaedic consultation) was permissible during follow-up.

All participants received follow-up questionnaires by post at two weeks, and then at two, four, and six months. 10 days after each timepoint a reminder was sent (at two weeks, a further questionnaire, and after two, four, and six months, a reminder postcard). At the two, four, and six month time points, people who had not responded to the postcard were sent another questionnaire after 10 days and then telephoned (by a masked research nurse) after a further 10 days. A brief questionnaire was posted to those who could not be contacted after five telephone attempts.

### Outcomes

The primary outcome was current hip pain intensity (hip pain today) reported by the patient, measured using a 0-10 Numerical Rating Scale over six months follow-up (through repeated measures at two weeks, and at two, four, and six months after randomisation).

The secondary outcome measures included pain, stiffness, and physical function (Western Ontario and McMaster University Osteoarthritis Index (WOMAC version 3.1 subscales)),^16^ participants’ self-reported global impression of change,^17^ general health (SF-12 and EuroQoL Q-5D-5L),^18^ ^19^ sleep disturbance (adapted from Dawson et al),^20^ pain self-efficacy,^21^ modified brief illness perceptions,^22^ maintenance of and return to desired activities such as work and social life, healthcare use including drug use and participant incurred costs, treatments received (including analgesia and referral for surgery), participants’ satisfaction and experience with the treatment, and work status (that is, employment status, presenteeism, absence). Receipt of other hip injections, adherence to exercise advice, and body mass index were assessed through follow-up questionnaires. The published protocol lists joint replacement surgery as a long term secondary outcome; this was not undertaken owing to lack of funding. Adverse events were self-reported and collected by participants’ general practitioner. Adverse events to an injection were collected by clinical case report forms and questionnaires (at two weeks and two months).

### Statistical analysis

For the primary comparison (BCT plus ultrasound-triamcinolone-lidocaine *v* BCT), originally, a sample size of 116 per group (348) was needed to provide 90% power (5% two-tailed significance) for superiority testing to detect at least a difference of 1.5 points in mean pain intensity (Numeric Rating Scale) score (anticipated standard deviation 4.5 points; standardised difference 0.33) with repeated-measures correlations of 0.50 and baseline-outcome of 0.33, and allowing for 15% loss to follow-up. On 27 March 2017, in response to suboptimal recruitment, the data monitoring committee advised investigating the validity of the parameters assumed in the original sample size calculation.^14^ The observed baseline standard deviation of the pain Numerical Rating Scale in participants recruited by this time point (n=65) was 1.7 and the standard deviation for follow-up scores was 2.5. At this point, we revised our target sample size to 68 in each group (204) to detect a minimum difference of 1 point in mean pain Numerical Rating Scale score (standard deviation 2.5; standardised difference 0.4) between BCT plus ultrasound-triamcinolone-lidocaine and BCT across the six month follow-up period (15% loss to follow-up, 80% power, 5% two tailed significance, and repeated measures and baseline-outcome correlations of 0.5 and 0.2, respectively).

Two blinded statisticians independently conducted analysis by intention to treat, following a pre-agreed statistical analysis plan. The main comparison was between BCT plus ultrasound-triamcinolone-lidocaine and BCT, with BCT plus ultrasound-triamcinolone-lidocaine versus BCT plus ultrasound-lidocaine a secondary comparison; all paired comparisons were undertaken before unblinding.

A linear mixed model for repeated measures was used to derive estimates of average (equally weighted overall follow-up) between group difference in pain Numerical Rating Scale scores across the four follow-up time points. The same model estimated differences in pain Numerical Rating Scale scores between groups at the two week, two month, four month, and six month time points through modelling the interaction of treatment group and (dummy) time. We used longitudinal mixed models to evaluate between group comparisons for the secondary outcome measures: using linear regression for numerical outcomes and log binomial regression (or Poisson regression with robust standard errors in the event of non-convergence) for categorical outcomes, with random effects at the patient level to take into account clustering through repeated measures.

Standardised mean differences were calculated as the ratio of the estimated mean difference to the standard deviation of pain scores (derived as the mean of standard deviations across follow-up times). We present absolute mean difference for numerical outcomes and relative risk, absolute risk difference, and number needed to treat (NNT) for binary outcomes. The pain Numerical Rating Scale was dichotomised around a cut-off of <5 (low pain) versus ≥5 (moderate to severe pain).

A sensitivity analysis of the primary outcome measure was performed through multiple imputation using chain equations with 20 imputations, inclusive of complete baseline variables and the baseline current pain Numerical Rating Scale score as predictors. We did multiple imputation analyses, incorporating plausible best-case and worst-case deviations from ignorable missingness,^23^ setting imputations as 1 point higher and lower than the imputed values (corresponding to the minimal clinically important difference of 1 point),^24^ and 2.5 points higher and lower (corresponding to the anticipated standard deviations for pain scores). We explored missing data patterns to examine association of missingness with baseline and follow-up measures; all variables were correlated using point-biserial correlation versus a created variable for full or incomplete follow-up data, and prognostic baseline characteristics were compared for those with full follow-up data between treatment groups to explore the effect of differential attrition. 

 We summarise the number and percentage of participants affected by protocol deviations. A per protocol subanalysis of the primary outcome was used, excluding trial treatment related deviators. Several prespecified exploratory subgroup analyses for the primary outcome were performed using linear mixed modelling, including an interaction term for treatment multiplied by baseline subgroup variable: participants receiving their preferred treatment (Yes/No), illness perceptions (split around baseline overall median value), body mass index (<25, ≥25), symptom duration (<six, ≥six months), pain severity (<5, ≥5),^25^ and, in the injection groups only, presence of synovitis or effusion (Yes/No). We report the numbers and percentages of adverse events, stratified by severity and by treatment group, including the number needed to harm for treatment comparisons.

All statistical tests were tests of superiority with 5% two tailed significance levels and adjusted for the following baseline covariates: eligibility current pain Numerical Rating Scale score, age, sex, and corresponding baseline value for secondary outcomes. The corresponding 95% confidence intervals were calculated. Statistical analyses were performed using STATA version 15 and R version 3.4.2. An external trial steering and data monitoring committee were appointed. 

### Patient and public involvement

A study patient advisory group advised on study design before funding, in study set-up, and during recruitment. They chose the term best current treatment and informed the design of clinic procedures (including how best to reduce the burden of intervention), questionnaire design, and participant information. This group informed protocol modifications in response to low recruitment, and guided interpretation of the findings. Two public contributors were members of the independent trial steering committee. 

## Results

**Fig 1:**
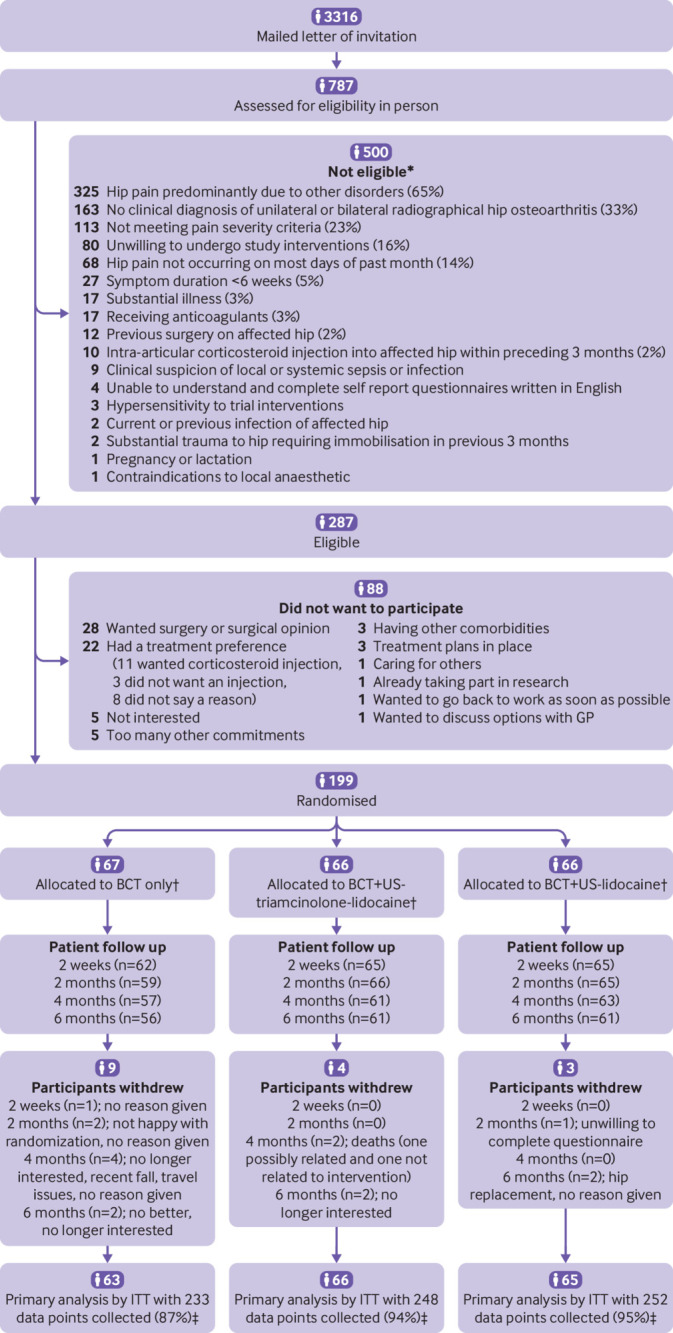
Trial flow chart. BCT=best current treatment. ITT=intention to treat. US=ultrasound. *Numbers do not tally to total because multiple reasons may have been recorded. †Protocol violations related to treatment were noted for eight participants: three for BCT, four for BCT-ultrasound-lidocaine, and one for BCT-ultrasound-triamcinolone-lidocaine (these were excluded from per protocol sensitivity analysis). Mean pain score immediately before withdrawal was 3.4. ‡Follow-up data available on at least one occasion. Of 741 follow-up responses, eight (1%) were missing primary outcome data: one for BCT, five for BCT-ultrasound-triamcinolone-lidocaine, and two for BCT-ultrasound-lidocaine.

Between 18 January 2016 and 21 May 2018, 3316 invitations were mailed out (general practice recruitment route only), 787 people were assessed for eligibility in person, and 199 participants were randomly assigned to an intervention group: 67 to BCT, 66 to BCT plus ultrasound-triamcinolone-lidocaine, and 66 to BCT plus ultrasound-lidocaine (fig 1). Baseline characteristics were similar between the groups (table 1), although in comparison with the other groups, the BCT group had more women and shorter pain duration and participants in the BCT plus ultrasound-triamcinolone-lidocaine group were more likely to have a paid job and less likely to have comorbidities or sleep disturbance. All participants received BCT according to the protocol. Overall, 32 (62%) of 52 participants receiving BCT agreed that they were adherent with exercises at two months, compared with 39 (64%) of 61 receiving BCT plus ultrasound-triamcinolone-lidocaine and 32 (53%) of 60 receiving BCT plus ultrasound-lidocaine, respectively (supplementary table 1). Six participants crossed over treatment group but were analysed in their randomised allocation group as per the intention-to-treat protocol. Three participants crossed over from BCT to BCT plus ultrasound-triamcinolone-lidocaine (between two and four months after randomisation), and three were not given the protocol treatment: one crossed over from BCT plus ultrasound-lidocaine to BCT, one from BCT plus ultrasound-triamcinolone-lidocaine to BCT, and one from BCT-ultrasound-lidocaine to BCT plus ultrasound-triamcinolone-lidocaine. Of the 199 participants, the primary outcome was completed by 188 (95%) participants at two weeks, 187 (94%) at two months, 179 (90%) at four months, and 178 (89%) at six months (average weighted follow-up rate across time points 93%). Little correlation was noted (that is, point-biserial correlation coefficient <0.3) between full or incomplete follow-up response and all baseline and follow-up variables. Sixteen participants withdrew (nine in the BCT group, three in the BCT plus ultrasound-lidocaine group, and four in the BCT plus ultrasound-triamcinolone-lidocaine group; fig 1).

**Table 1:** Baseline personal and clinical characteristics of trial participants. Values are numbers (percentages) unless stated otherwise

Characteristics	All (n=199)			BCT (n=67)		BCT+ultrasound-triamcinolone-lidocaine* (n=66)			BCT+ultrasound-lidocaine* (n=66)
Mean (SD) age (years)	62.8 (10.0)		63.7 (10.9)				62.5 (9.3)		62.3 (9.8)
Women	113 (57)		42 (63)				35 (53)		36 (55)
White ethnicity	198 (99)		67 (100)				66 (100)		65 (98)
Live alone	32 (16)		10 (15)				9 (14)		13 (20)
Employment status*:									
Paid work	88 (45)		25 (39)				37 (56)		26 (40)
Retired	82 (42)		30 (46)				23 (35)		29 (45)
No paid work	26 (13)		10 (15)				6 (9)		10 (15)
Mean (SD) body mass index	29.1 (5.8)		29.6 (6.7)				29.5 (5.6)		28.4 (4.9)
Smoking status:									
Never	97 (49)		33 (49)				35 (53)		29 (44)
Former	69 (35)		24 (36)				21 (32)		24 (36)
Currently	33 (16)		10 (15)				10 (15)		13 (20)
Alcohol intake:									
Daily or most days	31 (16)		12 (18)				12 (18)		7 (11)
Once or twice weekly	78 (39)		22 (33)				25 (38)		31 (47)
Once or twice monthly	36 (18)		16 (24)				11 (17)		9 (14)
Once or twice yearly	27 (14)		7 (10)				12 (18)		8 (12)
Never	27 (14)		10 (15)				6 (9)		11 (17)
Hips affected:									
Both	49 (25)		16 (24)				14 (21)		19 (29)
Right	90 (45)		32 (48)				28 (42)		30 (46)
Left	60 (30)		19 (28)				24 (36)		17 (26)
Duration of symptoms†:									
<3 months	6 (3)		2 (3)				3 (5)		1 (2)
3-6 months	17 (9)		8 (12)				5 (8)		4 (6)
6-12 months	42 (21)		19 (28)				9 (14)		14 (21)
>1 year	133 (67)		38 (57)				48 (74)		47 (71)
Days of pain in past 12 months:	1 (1)		0 (0)				0 (0)		1 (0)
<7 days	0 (0)		0 (0)				0 (0)		0 (0)
1-4 weeks	12 (6)		4 (6)				6 (9)		2 (3)
1-3 months	186 (93)		63 (94)				60 (91)		63 (96)
>3 months									
Previous injury:									
No	172 (86)		59 (89)				58 (88)		55 (83)
Right hip	11 (6)		2 (3)				4 (6)		5 (8)
Left hip	10 (5)		2 (3)				3 (5)		5 (8)
Both hips	5 (3)		3 (5)				1 (2)		1 (2)
Sleep disturbance:									
No nights	9 (5)		6 (9)				1 (2)		2 (3)
1-2 nights	10 (5)		2 (3)				3 (5)		5 (8)
Some nights	39 (20)		19 (28)				10 (15)		10 (15)
Most nights	62 (31)		19 (28)				24 (36)		19 (29)
Every night	79 (40)		21 (31)				28 (42)		30 (46)
Site of previous steroid injection:									
Hip	7 (4)		3 (4)				1 (2)		3 (5)
Other joints†	67 (34)		19 (28)				25 (38)		23 (35)
Preference for hip injection*	185 (94)		62 (93)				62 (95)		61 (95)
Received injection or not as preference*	128 (65)		5 (7)				62 (95)		61 (95)
Presence of effusion‡	—		—				9 (14)		9 (14)
Presence of synovitis‡	—		—				27 (42)		26 (40)
Comorbidity (other conditions)‡	129 (65)		47 (70)				36 (55)		46 (71)
Pain Numerical Rating Scale score (0-10):									
Mean (SD)	5.7 (2.1)		5.7 (2.2)				5.8 (2.1)		5.7 (2.1)
Median (IQR)	6 (4-8)		5 (4-8)				5 (4-8)		5 (4-8)
Mean (SD) WOMAC scores:									
Total	50.7 (15.6)		51.1 (19.0)				50.2 (14.8)		50.7 (13.0)
Pain	10.7 (3.3)		10.7 (4.0)				10.7 (2.8)		10.7 (3.2)
Stiffness	4.5 (1.5)		4.3 (1.5)				4.6 (1.4)		4.6 (1.5)
Function	35.5 (12.4)		36.0 (14.6)				35.0 (11.6)		35.4 (10.9)
Mean (SD) PSEQ	36.7 (13.7)		35.7 (14.7)				36.4 (13.4)		38.5 (13.0)
Mean (SD) IPQ scores:									
Total	30.1 (6.6)		30.7 (7.2)				29.8 (7.3)		29.9 (5.4)
Consequences	6.5 (2.1)		6.5 (2.4)				6.4 (2.1)		6.5 (1.8)
Timeline	9.0 (1.5)		9.0 (1.4)				8.7 (1.8)		9.2 (1.3)
Personal control	4.0 (2.7)		3.6 (2.8)				4.2 (2.6)		4.1 (2.9)
Treatment control	7.4 (2.0)		7.4 (2.0)				7.3 (2.0)		7.4 (2.0)
Emotional response	5.9 (2.8)		6.1 (2.9)				6.1 (2.7)		5.7 (2.8)
Mean (SD) EQ5D utility score	0.49 (0.23)		0.50 (0.22)				0.49 (0.23)		0.48 (0.24)
Mean (SD) SF-12 scores:									
Physical component	33.9 (9.0)		33.9 (9.1)				34.5 (9.0)		33.2 (8.9)
Mental component	51.2 (12.0)		49.3 (13.3)				51.5 (12.2)		52.9 (10.0)
Mean (SD) GAD-7	5.8 (6.0)		6.1 (6.3)				5.9 (5.9)		5.3 (5.8)
Mean (SD) PHQ-8	6.6 (6.6)		6.9 (6.2)				6.7 (6.2)		6.2 (6.0)
Mean (SD) SPS	20.1 (4.9)		20.0 (6.1)				20.2 (4.7)		20.2 (3.7)
Mean (SD) work performance	4.6 (2.8)		3.9 (3.3)				4.7 (2.4)		5.2 (2.8)
Support for previous hip problem:									
Advice on weight loss	45 (23)		17 (25)				17 (26)		11 (17)
Written information about hip pain or osteoarthritis	66 (33)		26 (39)				28 (42)		12 (18)
Advice on exercise	90 (45)		26 (39)				35 (53)		29 (44)
Referral to physiotherapy	82 (41)		29 (43)				30 (46)		23 (35)
Referral to rheumatology	19 (10)		12 (18)				3 (5)		4 (6)
Referral to orthopaedics	13 (7)		4 (6)				3 (5)		6 (9)
Referral to pain management	15 (8)		6 (9)				3 (5)		6 (9)
Painkillers on prescription	125 (63)		38 (57)				45 (68)		42 (64)
Walking aid	29 (15)		10 (15)				10 (15)		9 (14)
None of above	35 (18)		13 (19)				13 (20)		9 (14)

BCT=best current treatment; SD=standard deviation; IQR=interquartile range; WOMAC=Western Ontario and McMaster Universities Osteoarthritis Index (total: 0=minimum problems, 96=maximum problems; pain: 0=no pain, 20=maximal pain; stiffness: 0=no stiffness, 8=most stiffness; function: 0=no difficulty, 68=most difficulty) PSEQ=Pain Self-Efficacy Questionnaire (0=no confidence, 60=highest confidence); IPQ=modified brief Illness Perceptions Questionnaire (total: 0=full understanding, 50=least understanding; consequences: 0=no affect at all, 10=severely affects life; timeline: 0=last very short time, 10=last forever; personal control: 0=no control, 10=extreme control; treatment control : 0=treatment no help, 10=treatment extremely helpful; emotional response: 0=not affected emotionally, 10=extremely affected emotionally); SF-12 (physical component scale: 0=worst physical health, 100=best physical health; mental component scale: 0=worst mental health, 100=best mental health); EQ5D-5L (−0.59=worst health utility, 1.00=best health utility); SPS=Stanford Presenteeism Scale (6=minimum ability, 30=maximum ability); work performance numerical rating scale (0=not affected, 10=unable to do job); GAD-7=Generalised Anxiety Disorder (0=no anxiety, 21=severe anxiety); PHQ-8=Patient Health Questionnaire depression scale (0=no depression, 24=severe depression).

* Three responses missing.

† One response missing.

‡ Two responses missing.

**Fig 2:**
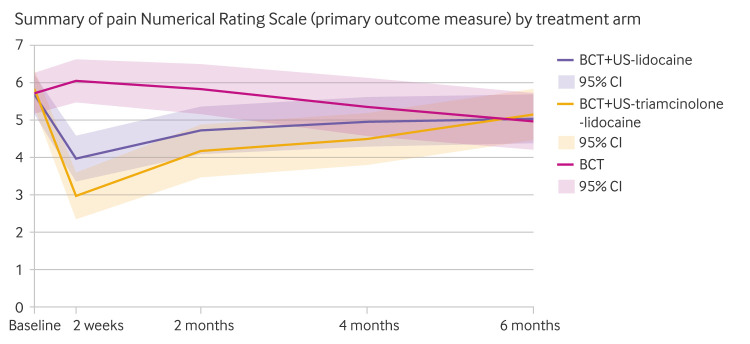
Summary of pain Numerical Rating Scale (primary outcome measure) by treatment group. BCT=best current treatment; US=ultrasound. An interactive version of this graphic is available at https://public.flourish.studio/visualisation/9137869/

Measured by the Numerical Rating Scale, a greater reduction was reported over six months in overall pain intensity in participants allocated to BCT plus ultrasound-triamcinolone-lidocaine compared with BCT: mean difference –1.43 (95% confidence interval −2.15 to −0.72), P<0.001; standardised mean difference −0.55 (−0.82 to −0.27; fig 2; supplementary table 2). A greater mean improvement was reported at two weeks (−3.17 (−4.06 to −2.28), P<0.001; −1.21, (−1.55 to −0.81)) and two months (−1.81 (−2.71 to −0.92), P<0.001; −0.69 (−1.03 to −0.35)), but not at four months (−0.86 (−1.78 to 0.05), P=0.06; −0.33 (−0.68 to 0.02)) or six months (0.12 (−0.80 to 1.04); P=0.80; −0.05 (−0.31 to 0.40)). Participants in the BCT plus ultrasound-triamcinolone-lidocaine group were more likely to meet the criterion for low pain (pain Numerical Rating Scale <5) at two weeks and at two and four months than were those in the BCT group (supplementary table 3). Participants in the BCT plus ultrasound-triamcinolone-lidocaine group were more likely to report feeling better and no sleep disturbance at two months than were those in the BCT group (relative risk 6.66 (95% confidence interval 2.48 to 17.85), NNT=3 and 1.96 (1.28 to 3.03), NNT=2). Unadjusted results are available in supplementary table 4.

Participants in the BCT plus ultrasound-triamcinolone-lidocaine arm compared with those in the BCT group had significantly greater overall improvement in pain and physical function (WOMAC, SF-12 physical component score), pain self-efficacy, illness perceptions, quality of life (EQ-5D-5L), and work presenteeism and performance (table 2). Generally, greater differences were observed at earlier follow-up time points (two weeks and two months) than at later time points (four and six months). More participants in the BCT plus ultrasound-triamcinolone-lidocaine arm reported being completely recovered or much better at two weeks (56% *v* 0%) and at two months (45% *v* 7%) than did participants with BCT; although, no significant percentage difference was reported at four months (27% *v* 17%; relative risk 1.54 (95% confidence interval 0.32 to 1.32); P=0.23, NNT=11) or six months (22% *v* 22%; 0.91 (0.55 to 2.17); P=0.79, NNT=–50). However, greater improvements in function and pain self-efficacy persisted to four months in the BCT-ultrasound-triamcinolone-lidocaine group compared with those receiving BCT (WOMAC function subscale −4.81 (−9.23 to −0.37), P=0.03; pain self-efficacy: 6.71 (2.51 to 10.92); table 2, supplementary table 5). Participants in the BCT plus ultrasound-triamcinolone-lidocaine group at six months were more likely to be satisfied with care and treatment received than those receiving BCT (58% *v* 34%; relative risk 1.72; (95% confidence interval 1.11 to 2.66), NNT=4) and were more likely to want the same care again (64% *v* 34%; 1.89 (1.22 to 2.92), NNT=3; table 3). Participants in the BCT plus ultrasound-triamcinolone-lidocaine group at two months were also more likely to report that they were not limited in their usual activities because of hip pain (67% *v* 45%; 1.79 (1.15 to 2.70), NNT=3).

**Table 2:** Summary of secondary health outcome measures by treatment group. Values are mean score (SD), number, unless stated otherwise

Outcome measures	BCT	BCT+ultrasound-triamcinolone-lidocaine	BCT+ultrasound-lidocaine	Effect size (95% CI)	
				BCT+ultrasound-triamcinolone-lidocaine *v* BCT	BCT+ultrasound-triamcinolone-lidocaine *v* BCT+ultrasound-lidocaine
**WOMAC**					
2 months	50.3 (21.1), 55	34.2 (20.3), 61	41.4 (19.2), 62	−14.77 (−20.91 to −8.64)*	−6.68 (−12.59 to −0.76)†
4 months	43.6 (23.1), 51	38.3 (20.7), 59	43.9 (18.5), 56	−6.38 (−12.54 to −0.21)†	−6.42 (−12.39 to −0.45)†
6 months	42.9 (22.6), 53	41.8 (20.8), 55	44.0 (19.4), 59	−1.42 (−7.68 to 4.84)	−0.78 (−6.82 to 5.27)
Overall	45.7 (22.4), 60	38.0 (20.7), 64	43.0 (19.0), 65	−7.52 (−13.00 to −2.04)‡	−4.62 (−9.91 to 0.67)
Subscales:					
Pain	9.6 (4.4)	7.9 (4.3)	9.0 (4.1)	−1.78 (−3.01 to −0.54)‡	−1.07 (−2.26 to 0.12)
Stiffness	3.9 (1.9)	3.5 (1.8)	3.8 (1.8)	−0.53 (−1.08 to 0.01)	−0.23 (−0.76 to 0.30)
Function	32.1 (16.7)	26.4 (15.1)	30.4 (14.0)	−5.47 (−9.41 to −1.53)‡	−3.60 (−7.40 to 0.21)
**PSEQ**					
2 months	34.3 (15.9), 55	44.4 (14.2), 62	39.2 (13.6), 63	9.27 (5.10 to 13.44)*	6.18 (2.15 to 10.20)‡
4 months	35.2 (16.7), 52	41.2 (15.0), 60	37.9 (13.2), 59	6.71 (2.51 to 10.92)‡	5.27 (1.21 to 9.34)†
6 months	37.8 (14.7), 53	38.8 (15.1), 58	36.9 (12.8), 59	1.64 (−2.62 to 5.90)	2.88 (−1.24 to 7.00)
Overall	35.7 (15.7), 60	41.5 (14.9), 65	38.0 (13.2), 65	5.87 (2.30 to 9.45)‡	4.78 (1.32 to 8.23)‡
**IPQ**					
2 months	33.0 (9.23), 51	27.2 (10.6), 60	30.0 (8.64), 60	−6.04 (−9.23 to −2.84)*	−2.55 (−5.59 to 0.48)
6 months	30.1 (9.16), 49	30.0 (9.03), 53	29.2 (9.49), 58	−0.15 (−3.44 to 3.14)	0.79 (−2.33 to 3.91)
Overall	31.6 (9.27), 58	28.5 (9.94), 63	29.6 (9.04), 65	−3.10 (−5.92 to −0.27)†	−0.88 (−3.57 to 1.80)
Subscales:					
Consequences	6.0 (2.6)	5.5 (2.7)	5.9 (2.3)	−0.55 (−1.27 to 0.17)	−0.35 (−1.04 to 0.35)
Timeline	8.7 (2.1)	8.7 (2.2)	8.4 (2.2)	0.07 (−0.74 to 0.60)	0.40 (−0.25 to 1.05)
Personal control	4.3 (2.7)	4.5 (2.9)	4.2 (2.7)	0.12 (−0.93 to 0.69)	0.25 (−0.52 to 1.03)
Treatment control	3.9 (2.9)	6.1 (3.2)	5.0 (3.1)	2.13 (1.11 to 3.15)*	0.98 (0.01 to 1.95)†
Emotional response	5.2 (2.9)	4.7 (3.1)	4.8 (2.9)	−0.63 (−1.43 to 0.16)	−0.43 (−1.19 to 0.33)
**SF-12 PCS**					
2 months	32.8 (8.0), 55	39.1 (9.8), 59	35.0 (9.5), 60	5.30 (2.38 to 8.21)*	4.05 (1.20 to 6.89)‡
4 months	35.7 (10.8), 53	39.0 (10.6), 58	33.7 (9.7), 53	3.07 (0.13 to 6.01)†	5.31 (2.41 to 8.21)*
6 months	33.7 (9.9), 50	37.7 (10.1), 51	34.0 (9.5), 54	3.04 (0 to 6.08)	2.15 (−0.81 to 5.11)
Overall	34.1 (9.6), 60	38.7 (10.1), 64	34.2 (9.6), 64	3.80 (1.33 to 6.27)‡	3.84 (1.43 to 6.24)‡
**SF-MCS**					
2 months	47.5 (12.9), 50	50.2 (12.0), 59	50.1 (12.3), 60	1.09 (−2.69 to 4.87)	−1.05 (−4.73 to 2.63)
4 months	46.3 (13.5), 53	49.4 (12.4), 58	49.9 (11.8), 53	2.01 (−1.80 to 5.83)	0.33 (−3.44 to 4.10)
6 months	49.8 (12.7), 50	48.7 (11.9), 51	49.2 (12.2), 54	−2.63 (−6.61 to 1.35)	−0.50 (−4.37 to 3.37)
Overall	47.8 (13.0), 60	49.5 (12.0), 64	49.8 (12.0), 64	0.16 (−2.83 to 3.15)	0.29 (−2.60 to 3.19)
**EQ5D-5L**					
2 weeks	0.47 (0.27), 61	0.64 (0.23), 63	0.59 (0.22), 64	0.18 (0.12 to 0.24)*	0.06 (0 to 0.12)
2 months	0.44 (0.29), 56	0.60 (0.26), 62	0.52 (0.24), 64	0.15 (0.08 to 0.22)*	0.07 (0 to 0.14)†
4 months	0.48 (0.28), 56	0.59 (0.23), 58	0.48 (0.28), 62	0.12 (0.04 to 0.19)‡	0.10 (0.03 to 0.18)‡
6 months	0.52 (0.25), 54	0.50 (0.25), 57	0.50 (0.24), 60	0.01 (−0.07 to 0.08)	0.00 (−0.07 to 0.08)
Overall	0.48 (0.27), 63	0.58 (0.25), 66	0.52 (0.25), 65	0.11 (0.06 to 0.17)*	0.06 (0.01 to 0.11)†
**SPS**					
2 months	20.0 (6.0), 22	23.4 (4.4), 35	20.1 (6.0), 27	4.06 (1.75 to 6.38)‡	3.40 (1.22 to 5.59)‡
6 months	19.9 (6.6), 21	21.8 (5.1), 32	20.0 (4.7), 26	2.16 (−0.20 to 4.53)	2.45 (0.22 to 4.69)†
Overall	19.9 (6.2), 43	22.7 (4.8), 67	20.1 (5.4), 53	3.11 (1.05 to 5.18)‡	2.93 (0.98 to 4.88)‡
**Work performance**					
2 months	4.1 (3.0), 21	3.1 (2.4), 32	4.9 (3.0), 28	−1.72 (−2.93 to −0.51)‡	−1.49 (−2.64 to −0.34)†
6 months	4.4 (3.2), 21	4.2 (2.7), 32	4.5 (2.7), 26	−0.84 (−2.08 to 0.39)	−0.47 (−1.64 to 0.71)
Overall	4.3 (3.1), 25	3.6 (2.6), 37	4.7 (2.8), 30	−1.28 (−2.39 to −0.18)†	−0.98 (−2.03 to 0.07)
**Body mass index**					
6 months	29.2 (6.1), 52	29.2 (5.4), 56	28.0 (4.7), 57	−0.22 (−0.91 to 0.46)	0.10 (−0.55 to 0.74)
**Perceived change (No (%))**					
2 weeks:					
Completely better§	0 (0)	2 (3)	1 (2)	RR=6.93 (3.39 to 14.17)* ¶	RR=2.28 (1.42 to 3.66)*
Much better§	0 (0)	34 (53)	15 (23)	RD=65 (26 to 100)	RD=31 (10 to 64)
Somewhat better	7 (11)	14 (22)	20 (31)	NNT=2 (1 to 4)	NNT=3 (2 to 10)
Same	39 (63)	10 (16)	23 (36)	—	—
Somewhat worse	10 (16)	4 (6)	4 (6)	—	—
Much worse	6 (10)	0 (0)	1 (2)	—	—
2 months:					
Completely better§	1 (2)	2 (3)	0 (0)	RR=6.66 (2.48 to 17.85)*	RR=2.63 (1.43 to 4.82)‡
Much better§	3 (5)	28 (42)	11 (17)	RD=40 (10 to 100)	RD=28 (7 to 65)
Somewhat better	6 (10)	9 (14)	21 (32)	NNT=3 (1 to 10)	NNT=4 (2 to 14)
Same	27 (47)	14 (21)	19 (29)	—	—
Somewhat worse	16 (28)	9 (14)	12 (19)	—	—
Much worse	5 (9)	4 (6)	2 (3)	—	—
4 months:					
Completely better§	0 (0)	1 (2)	0 (0)	RR=1.54 (0.32 to 1.32)	RR=1.85 (0.88 to 3.88)
Much better§	10 (17)	15 (25)	9 (14)	RD=9 (−12 to 5)	RD=12 (−2 to 40)
Somewhat better	5 (9)	15 (25)	10 (16)	NNT=11 (20 to −8)	NNT=8 (−50 to 3)
Same	17 (30)	14 (23)	24 (38)	—	—
Somewhat worse	16 (28)	13 (22)	14 (22)	—	—
Much worse	9 (16)	2 (3)	6 (10)	—	—
6 months:					
Completely better§	2 (4)	1 (2)	0 (0)	RR=0.91 (0.55 to 2.17)	RR=1.27 (0.62 to 2.58)
Much better§	10 (18)	13 (21)	11 (18)	RD=−2 (−10 to 26)	RD=5 (−7 to 28)
Somewhat better	7 (12)	13 (21)	12 (20)	NNT=−50 (4 to −10)	NNT=20 (4 to −14)
Same	14 (25)	`13 (21)	18 (29)	—	—
Somewhat worse	14 (25)	18 (30)	15 (25)	—	—
Much worse	9 (16)	3 (5)	5 (8)	—	—
**Sleep disturbance**					
2 months:					
No nights§	4 (7)	12 (19)	7 (11)	RR=1.96 (1.28 to 3.03)‡	RR=1.72 (1.12 to 2.63)†
1-2 nights§	5 (9)	16 (25)	5 (8)	RD=45 (13 to 95)	RD=36 (6 to 82)
Some nights§	17 (31)	15 (24)	19 (31)	NNT=2 (1 to 8)	NNT=3 (1 to 17)
Most nights	15 (27)	12 (19)	17 (27)	—	—
Every night	14 (25)	8 (13)	14 (23)	—	—
4 months:					
No nights§	8 (15)	9 (15)	6 (10)	RR=1.27 (0.79 to 2.04)	RR=1.56 (1.06 to 2.27)†
1-2 nights§	9 (17)	13 (22)	9 (15)	RD=17 (−13 to 64)	RD=25 (3 to 57)
Some nights§	16 (30)	15 (25)	12 (20)	NNT=6 (2 to −7)	NNT=4 (2 to 33)
Most nights	13 (24)	14 (24)	25 (41)	—	—
Every night	8 (15)	8 (14)	9 (15)	—	—
6 months:					
No nights§	9 (17)	8 (14)	7 (12)	RR=1.05 (0.69 to 1.45)	RR=1.10 (0.79 to 1.52)
1-2 nights§	6 (12)	5 (9)	5 (8)	RD=3 (−18 to 26)	RD=5 (−10 to 24)
Some nights§	15 (29)	14 (25)	16 (27)	NNT=33 (4 to −6)	NNT=20 (4 to −10)
Most nights	15 (29)	15 (26)	21 (35)	—	—
Every night	7 (13)	15 (26)	11 (18)	—	—

SD=standard deviation; BCT=best current treatment; CI=confidence interval; RR=relative risk; RD=percentage risk difference (derived as product of RR and observed positive proportion in BCT group (rounded to nearest integer and converted to percentage) minus observed positive percentage in BCT group); NNT=number needed to treat (=100/RD (rounded to nearest integer)).

Relative risk (RR) is shown for dichotomised outcomes. P values are analyses by linear or generalised mixed models accounting for repeated measures and adjusted for age, sex, baseline pain intensity, and (when applicable) corresponding baseline value.

WOMAC=Western Ontario and McMaster Universities Osteoarthritis Index (total: 0=minimum problems, 96=maximum problems; pain: 0=no pain, 20=maximal pain; stiffness: 0=no stiffness, 8=most stiffness; function: 0=no difficulty, 68=most difficulty) PSEQ=Pain Self-Efficacy Questionnaire (0=no confidence, 60=highest confidence); IPQ=modified brief Illness Perceptions Questionnaire (total: 0=full understanding, 50=least understanding; consequences: 0=no affect at all, 10=severely affects life; timeline: 0=last very short time, 10=last forever; personal control: 0=no control, 10=extreme control; treatment control : 0=treatment no help, 10=treatment extremely helpful; emotional response: 0=not affected emotionally, 10=extremely affected emotionally); SF-12 (physical component scale: 0=worst physical health, 100=best physical health; mental component scale: 0=worst mental health, 100=best mental health); EQ5D-5L (−0.59=worst health utility, 1.00=best health utility); SPS=Stanford Presenteeism Scale (6=minimum ability, 30=maximum ability); work performance numerical rating scale (0=not affected, 10=unable to do job).

* P<0.001.

† P=0.01 to <0.05.

‡ P=0.001 to <0.01.

§ For comparative analysis of categorical variables (derivation of RR), categories that were used to define a positive response coded as 1 (other categories denoting a negative response coded 0) according to preagreed rules for dichotomisation of categorical variables.

¶ RR was derived using completely/much/somewhat better as positive response since the number of people who were completely or much better at two weeks in the BCT group was zero and could not be analysed.

**Table 3:** Satisfaction and understanding end expectations of care by treatment group. Values are numbers (percentages) unless stated otherwise

Outcome measures	BCT	BCT+ultrasound-triamcinolone-lidocaine	BCT+ultrasound-lidocaine	Effect size (95% CI)	
				BCT+ultrasound-triamcinolone-lidocaine *v* BCT	BCT+ultrasound-triamcinolone-lidocaine *v* BCT-ultrasound-lidocaine
**Rating of overall results of care** *					
2 months	5 (3), n=50	7 (3), n=57	6 (3), n=61	2.51 (1.40 to 3.61)†	1.43 (0.38 to 2.47)‡
6 months	5 (3), n=51	7 (3), n=55	6 (3), n=58	1.60 (0.50 to 2.71)‡	0.15 (−0.91 to 1.21)
**Satisfaction with information received**					
2 months:					
Very satisfied§	15 (27)	35 (56)	28 (46)	RR=1.35 (1.10 to 1.64)‡	RR=1.15 (0.99 to 1.34)
Quite satisfied§	23 (41)	21 (34)	20 (33)	RD=24 (7 to 44)	RD=12 (−1 to 27)
No opinion	5 (9)	1 (2)	5 (8)	NNT=5 (2 to 15)	NNT=8 (4 to −100)
Not very satisfied	6 (11)	3 (5)	7 (11)		
Not at all satisfied	7 (12)	2 (3)	1 (2)		
6 months:					
Very satisfied§	12 (23)	23 (40)	29 (48)	RR=1.19 (0.91 to 1.56)	RR=1.15 (0.94 to 1.40)
Quite satisfied§	20 (39)	19 (33)	21 (35)	RD=12 (−6 to 35)	RD=16 (−5 to 33)
No opinion	8 (15)	8 (14)	6 (10)	NNT=9 (3 to −17)	NNT=6 (3 to −20)
Not very satisfied	9 (17)	6 (10)	4 (7)		
Not at all satisfied	3 (6)	2 (3)	0 (0)		
**Understanding of hip problem**					
2 months:					
Very clear§	19 (34)	25 (40)	29 (47)	RR=1.01 (0.87 to 1.17)	RR=1.02 (0.89 to 1.17)
Quite clear§	29 (52)	29 (47)	24 (39)	RD=1 (−11 to 14)	RD=2 (−10 to 15)
No opinion	2 (4)	1 (2)	5 (8)	NNT=100 (8 to −9)	NNT=50 (7 to −10)
Not very clear	6 (11)	7 (11)	4 (6)		
6 months:					
Very clear§	18 (35)	22 (38)	24 (40)	RR=0.98 (0.81 to 1.18)	RR=0.88 (0.76 to 1.03)
Quite clear§	24 (46)	24 (41)	30 (50)	RD=−2 (−15 to 15)	RD=−11 (−22 to 3)
No opinion	5 (10)	5 (9)	1 (2)	NNT=−50 (7 to −7)	NNT=−9 (33 to −5)
Not very clear	5 (10)	7 (12)	5 (8)		
**Patient still has questions about their hip problem**					
2 months:				RR=1.19 (0.76 to 1.82)	RR=1.16 (0.76 to 1.75)
No§	30 (55)	37 (61)	33 (54)	RD=10 (−13 to 45)	RD=9 (−13 to 41)
Yes	25 (45)	24 (39)	28 (46)	NNT=10 (2 to −8)	NNT=11 (2 to −8)
6 months:				RR=0.96 (0.65 to 1.43)	RR=1.14 (0.80 to 1.61)
No§	27 (52)	28 (49)	26 (43)	RD=−2 (−18 to 22)	RD=6 (−9 to 26)
Yes	25 (48)	29 (51)	34 (57)	NNT=−50 (5 to −6)	NNT=17 (4 to −11)
**Patient has been kept from their usual activities because of hip pain**					
2 months:					
No§	25 (45)	40 (67)	32 (52)	RR=1.79 (1.15 to 2.70)‡	RR=1.41 (0.91 to 2.22)
Yes	31 (55)	20 (33)	30 (48)	RD=36 (7 to 77)	RD=21 (−5 to 63)
If yes, how prepared patient feels about returning to normal activities and work:				NNT=3 (1 to 14)	NNT=5 (2 to −20)
Very prepared	1	9	1		
Quite prepared	8	11	9		
No opinion	15	8	7		
Not very prepared	10	10	14		
Not at all prepared	4	2	4		
6 months:					
No§	25 (47)	27 (49)	28 (47)	RR=1.15 (0.79 to 1.69)	RR=1.08 (0.76 to 1.54)
Yes:	28 (53)	28 (51)	31 (53)	RD=7 (−10 to 32)	RD=4 (−11 to 25)
Very prepared	3	5	4	NNT=14 (3 to −10)	NNT=25 (4 to −9)
Quite prepared	8	6	11		
No opinion	17	10	12		
Not very prepared	7	12	9		
Not at all prepared	4	4	5		
**Satisfaction with care received**					
2 months:					
Very satisfied§	6 (12)	27 (47)	14 (23)	RR=1.97 (1.33 to 2.93)‡	RR=1.61 (1.17 to 2.21)‡
Quite satisfied§	13 (25)	14 (25)	14 (23)	RD=36 (12 to 71)	RD=28 (8 to 56)
No opinion	15 (29)	9 (16)	22 (36)	NNT=3 (1 to 8)	NNT=4 (2 to 13)
Not very satisfied	10 (20)	5 (9)	7 (11)		
Not at all satisfied	7 (14)	2 (3)	5 (8)		
6 months:					
Very satisfied§	6 (11)	15 (27)	17 (30)	RR=1.72 (1.11 to 2.66)¶	RR=0.93 (0.69 to 1.25)
Quite satisfied§	12 (23)	17 (31)	19 (33)	RD=24 (4 to 56)	RD=−4 (−20 to 16)
No opinion	20 (39)	12 (22)	15 (26)	NNT=4 (2 to 25)	NNT=−25 (6 to −5)
Not very satisfied	10 (19)	7 (13)	6 (11)		
Not at all satisfied	4 (8)	4 (7)	0 (0)		
**Patient would have same care again if they had same condition**					
2 months:					
Definitely§	4 (8)	26 (45)	16 (26)	RR=2.41 (1.53 to 3.80)†	RR=1.45 (1.07 to 1.96) ¶
Probably§	11 (22)	15 (26)	14 (23)	RD=42 (16 to 84)	RD=22 (3 to 47)
No opinion	16 (31)	11 (19)	14 (23)	NNT=2 (1 to 6)	NNT=5 (2 to 33)
Probably not	12 (23)	4 (7)	11 (18)		
Definitely not	8 (16)	2 (3)	6 (10)		
6 months:					
Definitely§	6 (12)	18 (33)	17 (29)	RR=1.89 (1.22 to 2.92)‡	RR=1.06 (0.80 to 1.40)
Probably§	11 (22)	17 (31)	19 (33)	RD=30 (7 to 65)	RD=4 (−12 to 25)
No opinion	20 (41)	10 (19)	10 (17)	NNT=3 (2 to 14)	NNT=25 (4 to −8)
Probably not	7 (14)	5 (9)	7 (12)		
Definitely not	5 (10)	4 (7)	5 (9)		
**Expectations for pain relief met**					
2 months:					
Probably met§	15 (28)	32 (56)	22 (35)	RR=1.52 (1.10 to 2.10)¶	RR=1.26 (0.95 to 1.67)
No opinion§	11 (21)	8 (14)	13 (21)	RD=25 (5 to 54)	RD=15 (−3 to 38)
Not met	20 (38)	12 (21)	19 (31)	NNT=4 (2 to 20)	NNT=7 (3 to −33)
Definitely not met	7 (13)	5 (9)	8 (13)		
6 months:					
Probably met§	17 (33)	25 (44)	22 (37)	RR=1.35 (0.99 to 1.84)	RR=1.29 (0.95 to 1.73)
No opinion§	10 (20)	13 (23)	9 (15)	RD=19 (−1 to 45)	RD=15 (−3 to 38)
Not met	15 (29)	13 (23)	20 (34)	NNT=5 (2 to −100)	NNT=7 (3 to −33)
Definitely not met	9 (18)	6 (10)	8 (14)		
Perceived treatment allocation (injection type)					
Unsure	—	54 (86)	50 (81)	—	—
Guessed correctly	—	9 (14)	2 (3)	—	—
Guessed incorrectly	—	0 (0)	10 (16)	—	—

SD=standard deviation; BCT=best current treatment; CI=confidence interval; RR=relative risk; RD=percentage risk difference (derived as product of RR and observed positive proportion in BCT group (rounded to nearest integer and converted to percentage) minus observed positive percentage in BCT group); NNT=number needed to treat (=100/RD (rounded to nearest integer)).

For numerical outcomes, effects shown are mean differences (summarised by mean (SD) scores).

P values were for analyses by linear or generalised mixed models accounting for repeated measures and adjusted for age, sex, baseline pain, and (when applicable) corresponding baseline value).

* Rating of overall results of care: 0-10 numerical integer scale (0=terrible, 10=excellent).

† P<0.001.

‡ P=0.001 to <0.01.

§ For comparative analysis of categorical variables (derivation of RR), categories that were used to define a positive response coded as 1 (other categories denoting a negative response coded 0) according to preagreed rules for dichotomisation of the categorical variables.

¶ P=0.01 to <0.05.

For the secondary comparison, the BCT plus ultrasound-triamcinolone-lidocaine versus BCT plus ultrasound-lidocaine mean difference between groups in pain was: −0.52 (95% confidence interval −1.21 to 0.18), P=0.15 overall; −1.02 (−1.90 to −0.14), P=0.02 at two weeks; −0.67 (−1.54 to 0.21), P=0.14 at two months; −0.48 (−1.37 to 0.41, P=0.29 at four months; and 0.10 (−0.79 to 1.00), P=0.82 at six months (fig 2; supplementary table 2). Significantly higher overall mean improvement for BCT plus ultrasound-triamcinolone-lidocaine over BCT plus ultrasound-lidocaine was observed for several secondary outcomes: pain self-efficacy, illness perceptions relating to treatment control, quality of life (EQ-5D-5L), physical function (WOMAC-F, SF-12-physical component scale), global impression of change, sleep disturbance, and work presenteeism and performance (table 2; supplementary table 5). Between group differences were generally highest at the earlier than later follow-up time points. Participants in the BCT plus ultrasound-triamcinolone-lidocaine arm were more likely to be satisfied with care and treatment received compared with participants in the BCT group, although no difference was recorded in whether expectations were met (table 3). No participant unblinding was evident with respect to injection type (table 3).

No unexpected adverse events were reported. In the BCT plus ultrasound-triamcinolone-lidocaine arm, four (6%) of 66 participants reported thinning or whitening of the skin at the injection site and four (6%) of 66 had hot flushes (number needed to harm 3.47 (95% confidence interval 2.39 to 6.54), supplementary table 6). Seven serious adverse events were recorded. One event was considered possibly related to trial treatment: a participant with a bioprosthetic aortic valve died from subacute bacterial endocarditis four months after receiving BCT plus ultrasound-triamcinolone-lidocaine. The participant had no signs of infection at randomisation. After discussion with the independent trial steering committee, data monitoring committee, and the participant’s treating cardiologist, we considered that the possibility of a causal link could not be excluded. The remainder were judged unrelated to the trial treatment (supplementary table 6).

Sensitivity analyses using multiple imputation gave similar results to the main analysis (supplementary table 7). Per protocol analysis (excluding the eight participants with treatment related protocol violations) gave a similar between arm difference over six months to the primary intention-to-treat result: mean difference −1.41 (95% confidence interval −2.14 to −0.67). A significant interaction effect was noted for the presence of synovitis or effusion on ultrasound where the between arm difference between injection groups favoured BCT plus ultrasound-triamcinolone-lidocaine if synovitis or effusion was present: mean difference −1.70; (−3.10 to −0.30). No other significant subgroup interactions for pain intensity were noted (supplementary table 8).

## Discussion

### Principal findings

An ultrasound guided intra-articular hip injection of triamcinolone acetonide and lidocaine hydrochloride combined with BCT (BCT plus ultrasound-triamcinolone-lidocaine) led to greater pain reduction and improvement in function over a six month period in adults with hip osteoarthritis. When individual time points were examined, the differences in pain and function in participants receiving BCT plus ultrasound-triamcinolone-lidocaine compared with those receiving BCT only, mostly occurred early (at two weeks and two months for pain and function and four months for function only), and no significant differences in pain or function were reported at the six month time point. We identified only known and expected adverse reactions, including one death from subacute bacterial endocarditis, which was deemed possibly related to trial treatment.

### Strengths and limitations of this study

The strengths of our trial include the large sample size, the inclusion of two comparison arms, the length of follow-up, and the high response rates. To optimise generalisability, we recruited from both primary care and community based musculoskeletal services and had pragmatic inclusion and exclusion criteria. Most participants (94%) preferred to receive an injection, meaning that individuals in the BCT group, which did not receive an injection, might have experienced resentful demoralisation that might have accentuated differences between arms. However, our subgroup analysis did not support an interaction between treatment preference and outcome. Self-reported outcomes are a limitation because the primary comparison of BCT versus BCT plus ultrasound-triamcinolone-lidocaine was unblinded. Participants and clinicians were blinded to injection type; nine (14%) participants in the BCT plus ultrasound-triamcinolone-lidocaine group identified their injection correctly (table 3), although most people (>80%) in both groups were unsure. Recruitment was challenging and necessitated amendments to the inclusion criteria and target sample size. Recruitment did not reach the revised target of 204 participants by five participants; however, the follow-up exceeded the required number of participants at the primary endpoint. The trial was powered for the primary comparison of BCT plus ultrasound-triamcinolone-lidocaine versus BCT, and the study was probably underpowered to assess differences between the two injection groups, and also to detect significant interactions between baseline moderators of treatment and outcomes. However, our primary interest in this pragmatic trial was effectiveness of BCT plus ultrasound-triamcinolone-lidocaine versus BCT, and including a BCT group enabled a cost effectiveness analysis (reported separately). We did not include a placebo comparison, such as saline; in our locality, corticosteroid hip injections are available within the National Health Service (despite the limited evidence base) and we felt that a placebo controlled trial evaluating a therapy that is available would not be ethically appropriate and might adversely affect patients’ willingness to participate. Loss to follow-up was higher in the BCT group; however, results from our sensitivity analysis accounting for missing data showed similar findings to the main analysis. Finally, we did not include radiographical outcomes. Participants were mostly of white ethnicity possibly limiting the generalisability of the findings to other ethnic groups. 

### Comparison with other studies

This study is the largest randomised controlled trial of the clinical effectiveness of intra-articular corticosteroid injection in hip osteoarthritis. A systematic review of previously published randomised controlled trials of corticosteroid injections in hip osteoarthritis reported that the quality of evidence was relatively poor. Pooled analysis of data from two of five trials (n=90) showed that participants with hip osteoarthritis treated with corticosteroid injection were eight times more likely than people treated with controls to meet the outcome measures in the response criteria for pain and function by Rheumatology Clinical Trials Osteoarthritis Research Society International at eight weeks after hip injection.^9^ Our trial shows that improvements in function are sustained for at least four months after injection.

Evidence from systematic reviews and an individual patient data meta-analysis showed that people with increased pain, but with no signs of inflammation, derived more benefit from corticosteroid injection in the hip and knee.^26^ ^27^ Our findings, consistent with those from a previous trial of hip osteoarthritis, suggest that the presence of synovitis or effusion on ultrasound might predict response in people with hip osteoarthritis.^13^ The presence of inflammation is possibly more important for hip rather than for knee injections.

### Possible implications for clinicians and policy makers

The differences in pain and function in participants receiving BCT plus ultrasound-triamcinolone-lidocaine compared with those receiving BCT only mostly occurred at early follow-up time points and were no longer clinically or statistically significant at the six month time point; nonetheless, our findings suggest intra-articular corticosteroid injection may lead to more longlasting effects compared with a consultation comprising information, advice, and education alone. Some benefits persisted at four months, which is longer than the therapeutic effect of triamcinolone. This could be explained by pain relief from the injection facilitating engagement with other core treatments and return to valued activities such as work, although this not supported by our self-reported data on exercise adherence and body mass index.

We found no significant difference between the injection groups in hip pain intensity over six months; although the trial was not powered for this comparison. However, we found significant, clinically important differences between the injection groups in secondary outcome measures (eg, pain self-efficacy at two months, SF-12 physical component score at over six months, and total WOMAC score at four months), which compare favourably with published clinically important differences,^28^ ^29^ ^30^ suggesting a difference in efficacy between the groups. Although lidocaine has a short half life, it has been postulated to have anti-inflammatory effects and has been shown to reduce pain for up to three months in a trial comparing three injections of lidocaine with three injections of saline administered at weekly intervals.^31^ Participants might also have benefited from contextual effects of the injection; previous research has identified contextual treatment effects as being greater in studies of injection treatments than in studies of other treatments because these effects are likely to be influenced by patient preferences for injection,^32^ which had a high number of patient preferences in this study. Only small numbers of participants in the BCT group reported improvement, suggesting this intervention was either ineffective or participants were resentful about not receiving the experimental intervention (resentful demoralisation). Although exercise has an established evidence base for hip osteoarthritis, improvement is based on individualised, progressed exercise rather than one session comprising information, advice, and education. Arguably one consultation cannot be defined as best current treatment; however, a single contact for advice about exercise and to give an injection reflects some current UK models of care for osteoarthritis.^33^

Comparison of the effectiveness and safety of administering corticosteroid by alternative routes needs further research, given that a randomised controlled trial in people with hip osteoarthritis showed that intramuscular glucocorticoid injection was effective in reducing pain up to 12 weeks.^34^ As imaging findings of inflammation seem to predict response to injection, future research could investigate the effectiveness, acceptability, and feasibility of a stratified approach to treatment. A need remains to understand more fully the incidence and risk factors for adverse reactions associated with intra-articular corticosteroid injections, such as infection.^35^ Furthermore, in view of recent studies suggesting that multiple corticosteroid injections might be associated with loss of cartilage volume or radiographical evidence of worsening of osteoarthritis,^36^ ^37^ further studies are needed to investigate the safety, effectiveness, and optimal timing of repeated hip injections.

### Conclusions

In community settings of musculoskeletal services, we have shown that an ultrasound guided intra-articular hip injection of corticosteroid and local anaesthetic, administered with advice and education, is a clinically effective treatment for rapid and sustained symptom response compared with advice and education alone for people with hip osteoarthritis. These findings provide evidence to inform international guidelines and support treatment decision making for policy makers, payers (commissioners), GPs, and clinicians in musculoskeletal services. Our patient advisory group felt that these findings offer an important choice to patients, particularly those who are unsuitable for surgery and might feel their treatment options are limited. As in routine clinical practice, caution should be applied in patients with risk factors for, or signs of, infection. Increased susceptibility to infection, greater severity of infection, and masking of symptoms and signs of infection are recognised after intra-articular corticosteroid injection. Our findings do not confirm or refute a causal link between glucocorticoid injection and bacterial endocarditis, but clinicians should specifically exert caution and carefully counsel patients with risk factors for endocarditis, such as those with a prosthetic heart valve.

**What is already known on this topic**

Evidence for the effectiveness of intra-articular corticosteroid injection for hip osteoarthritis comprises five small trials of short duration in participants with severe osteoarthritis
Previous trials have shown clinical benefit eight weeks after hip injection in participants with hip osteoarthritis

**What this study adds**

In patients with mild to moderate hip osteoarthritis, ultrasound guided corticosteroid and local anaesthetic injection with advice and education led to greater pain reduction and improvement in function over six months, compared with advice and education alone
These findings provide evidence to inform international guidelines and offer important choice to patients, who often believe their treatment options are limited

## Data Availability

Data for this study will be made available to the scientific community on request after publication. Data will be made available for scientific purposes for researchers whose proposed use of the data has been approved by a publication committee. Data and documentation will be made available through a secure file exchange platform after approval of proposal and a data transfer agreement is signed (which defines obligations that the data requester must adhere to regarding privacy and data handling). Partially deidentified participant data limited to the data used for this work will be made available. For data access, please contact the corresponding author.

## Web extra

Extra material supplied by authors

Web appendix: Supplementary tables 1-8

## Notes

Contributors: All authors contributed to trial design, interpretation of data, and critical revision of the paper; approved the final version of the report; and agree to be accountable for all aspects of the work. CDM, ER, GP, CJ, and NEF conceived the trial. ZP, ER, AM, AH, CJ, NEF, RO, GP, KS, and CDM secured funding and wrote the protocol. ZP, ER, GH, EH, AC, SH, AH, AM, PR, and KS contributed to acquisition of trial data. MAH and CJ led the linked qualitative study. KB and ML analysed the clinical outcomes. RO and JK undertook the health economic analysis. ZP wrote the first draft of the paper with input from ER, KB, RO, and ML. ZP, ER, and CDM accept full responsibility for the work and the conduct of the study, had access to the data, and controlled the decision to publish. The corresponding author attests that all listed authors meet authorship criteria and that no others meeting the criteria have been omitted.

The senior author (ER) affirms that the manuscript is an honest, accurate, and transparent account of the study being reported; that no important aspects of the study have been omitted; and that any discrepancies from the study as originally planned (and, if relevant, registered) have been explained.

Dissemination to participants and related patient and public communities: The findings of this trial are being disseminated to clinicians and patients via the participating centres, websites of the authors’ institutes, as well as national and international rheumatology and osteoarthritis conferences. A plain language summary will be written to make the findings accessible to the public. The results will be shared on social media by the authors. Participants will receive a letter notifying them of the results and their treatment allocation.

Provenance and peer review: Not commissioned; externally peer reviewed.
