# Supplementary material for: Clinical effectiveness of one ultrasound guided intra-articular corticosteroid and local anaesthetic injection in addition to advice and education for hip osteoarthritis (HIT trial): single blind, parallel group, three arm, randomised controlled trial
Source: BMJ. 2022 Apr 6;377:e068446. doi: 10.1136/bmj-2021-068446 (PMC8984871; doi:10.1136/bmj-2021-068446)
Supplement: Supplementary file 1 — Web appendix: Supplementary tables 1-8 [file pasz068446.ww.pdf]

Supplementary Table 1: Summary of adherence to the BCT advice and education about exercise.

	BCT	BCT-US-Triamcinolone-Lidocaine	BCT-US-Lidocaine
Did read information leaflets given during clinic visit	54/67 (81%)	63/66 (95%)	62/66 (94%)
Doing exercises as often as advised to			
2 months			
Strongly agree / Agree	32 (62%)	39 (64%)	32 (53%)
Not sure	7 (13%)	7 (12%)	10 (17%)
Strongly disagree / Disagree	13 (25%)	15 (25%)	18 (30%)
4 months			
Strongly agree / Agree	29 (57%)	29 (49%)	28 (48%)
Not sure	9 (18%)	13 (22%)	11 (19%)
Strongly disagree / Disagree	13 (25%)	17 (29%)	19 (33%)
6 months			
Strongly agree / Agree	28 (54%)	28 (49%)	30 (50%)
Not sure	7 (13%)	11 (19%)	8 (13%)
Strongly disagree / Disagree	17 (33%)	18 (32%)	22 (37%)
Number of times a week doing exercises			
2 months			
Never	4 (8%)	6 (10%)	3 (5%)
1 – 2 times	15 (29%)	20 (32%)	23 (37%)
3 – 4 times	18 (35%)	24 (39%)	21 (34%)
5 – 6 times	5 (10%)	3 (5%)	3 (5%)
Every day	10 (19%)	9 (14%)	12 (19%)
4 months			
Never	12 (24%)	6 (10%)	5 (8%)
1 – 2 times	13 (25%)	23 (40%)	23 (39%)
3 – 4 times	17 (33%)	17 (29%)	19 (32%)
5 – 6 times	4 (8%)	5 (9%)	4 (7%)
Every day	5 (10%)	7 (12%)	8 (14%)
6 months			
Never	6 (11%)	4 (7%)	6 (10%)
1 – 2 times	17 (32%)	32 (56%)	25 (42%)
3 – 4 times	16 (30%)	10 (18%)	19 (32%)
5 – 6 times	9 (17%)	4 (7%)	2 (3%)
Every day	5 (9%)	7 (12%)	7 (12%)
Duration (minutes) exercising			
2 months			
< 5 minutes	2 (4%)	4 (7%)	5 (9%)
5 – 9 minutes	10 (20%)	16 (29%)	14 (24%)
10 – 14 minutes	17 (34%)	17 (30%)	22 (38%)
15 – 29 minutes	16 (32%)	17 (30%)	14 (24%)
≥ 30 minutes	5 (10%)	2 (4%)	3 (5%)
4 months			
< 5 minutes	3 (7%)	6 (11%)	3 (5%)
5 – 9 minutes	13 (31%)	16 (30%)	29 (52%)
10 – 14 minutes	14 (33%)	16 (30%)	11 (20%)
15 – 29 minutes	8 (19%)	12 (22%)	10 (18%)
≥ 30 minutes	4 (10%)	4 (7%)	3 (5%)
6 months			
< 5 minutes	0 (0%)	3 (6%)	5 (9%)
5 – 9 minutes	7 (17%)	19 (35%)	14 (26%)
10 – 14 minutes	17 (40%)	14 (26%)	21 (39%)
15 – 29 minutes	15 (36%)	14 (26%)	9 (17%)

≥ 30 minutes	3 (7%)	4 (7%)	5 (9%)

Supplementary Table 2: Summary of pain NRS (primary outcome measure) by treatment arm

	BCT	BCT-US-Triamcinolone-Lidocaine	BCT-US-Lidocaine	BCT-US-Triamcinolone-Lidocaine v BCT	BCT-US-Triamcinolone-Lidocaine v BCT-US-Lidocaine
	mean (SD), n	*mean (SD), n*	*mean (SD), n*	*mean difference (95% CI)* {SMD (95% CI)} [P value]*	*mean difference (95% CI)* {SMD (95% CI)} [P value]*
**2 weeks**	6.0 (2.3), 62	3.0 (2.5), 63	4.0 (2.4), 63	-3.17 (-4.06, -2.28) {-1.21 (-1.55, -0.81)} [P<0.001]	-1.02 (-1.90, -0.14) {-0.39 (-0.73, -0.05)} [P=0.023]
**2 months**	5.8 (2.5), 58	4.2 (2.8), 64	4.7 (2.6), 65	-1.81 (-2.71, -0.92) {-0.69 (-1.03, -0.35)} [P<0.001]	-0.67 (-1.54, 0.21) {-0.25 (-0.59, 0.08)} [P=0.136]
**4 months**	5.4 (2.9), 57	4.5 (2.7), 59	5.0 (2.6), 63	-0.86 (-1.78, 0.05) {-0.33 (-0.68, 0.02)} [P=0.063]	-0.48 (-1.37, 0.41) {-0.18 (-0.52, 0.16)} [P=0.291]
**6 months**	5.0 (2.8), 56	5.1 (2.7), 61	5.0 (2.5), 61	0.12 (-0.80, 1.04) {0.05 (-0.31, 0.40)} [P=0.797]	0.10 (-0.79, 1.00) {0.04 (-0.30, 0.38)} [P=0.823]
**Overall (average)**	5.6 (2.7), 63	4.2 (2.8), 66	4.7 (2.6), 65	-1.43 (-2.15, -0.72)[#1] {-0.55 (-0.82, -0.27)} [P<0.001]	-0.52 (-1.21, 0.18)[#2] {-0.20 (-0.46, 0.07)} [P=0.148]

Supplementary Table 3: Relative risk, risk difference and number need to treat for dichotomised pain intensity NRS (pain score <5, ≥5)

	BCT-US-Triamcinolone-Lidocaine v BCT	BCT-US-Triamcinolone-Lidocaine v BCT-US-Lidocaine
2 weeks		
- RR (95% CI)	2.73 (1.75 to 4.26) [P<0.001][3]	1.37 (1.03 to 1.82) [P=0.03][1]
- RD (95% CI)	0.45 (0.19 to 0.84)	0.19 (0.02 to 0.42)
- NNT (95% CI)	2.2 (1.2 to 5.3)	5.3 (2.4 to 50)
2 months		
- RR (95% CI)	1.75 (1.16 to 2.63) [P=0.007][2]	1.20 (0.87 to 1.65) [P=0.28]
- RD (95% CI)	0.26 (0.06 to 0.56)	0.10 (-0.06 to 0.32)
- NNT (95% CI)	3.8 (1.8 to 17)	10 (-17 to 3.1)
4 months		
- RR (95% CI)	1.63 (1.05 to 2.52) [P=0.03][1]	1.24 (0.85 to 1.80) [P=0.26]
- RD (95% CI)	0.21 (0.02 to 0.51)	0.10 (-0.06 to 0.34)
- NNT (95% CI)	4.8 (2.0 to 50)	10 (-17 to 2.9)
6 months		
- RR (95% CI)	0.99 (0.66 to 1.50) [P=0.97]	1.10 (0.72 to 1.70) [P=0.66]
- RD (95% CI)	0.00 (-0.15 to 0.21)	0.04 (-0.11 to 0.26)
- NNT (95% CI)	∞ (-6.7 to 4.8)	25 (-9.1 to 3.8)

BCT = Best Current Treatment; BCT-US-Lidocaine = BCT plus Ultrasound guided injection of lidocaine only; BCT-US-Triamcinolone-Lidocaine = BCT plus Ultrasound guided injection of triamcinolone and lidocaine; RR = Relative risk (Risk$_{\text{BCT-US-Triamcinolone-Lidocaine}}$/Risk$_{\text{BCT}}$); RD = Risk difference (Risk$_{\text{BCT-US-Triamcinolone-Lidocaine}}$ - Risk$_{\text{BCT}}$); NNT = Number Needed to Treat (US-T versus BCT)

[1] 0.01<P<0.05; [2] 0.001<P<0.01; [3] P<0.001 (analyses by generalized mixed model (with log link function) adjusted for age, gender and baseline pain score).

Supplementary Table 4: Adjusted versus unadjusted analysis of primary and secondary outcomes

	Adjusted analysis		*Unadjusted analysis*	
	**BCT-US-Triamcinolone-Lidocaine v BCT**	**BCT-US-Triamcinolone-Lidocaine v BCT-US-Lidocaine**	**BCT-US-Triamcinolone-Lidocaine v BCT**	**BCT-US-Triamcinolone-Lidocaine v BCT-US-Lidocaine**
**Primary outcome - Pain**				
2 weeks	-3.17 (-4.06 to -2.28)[1]	-1.02 (-1.90 to -0.14)[3]	-3.08 (-3.99 to -2.18)[3]	-0.98 (-1.88 to -0.07)[1]
2 months	-1.81 (-2.71 to -0.92)[3]	-0.67 (-1.54 to 0.21)	-1.72 (-2.62 to -0.80)[3]	-0.62 (-1.52 to 0.27)
4 months	-0.86 (-1.78 to 0.05)	-0.48 (-1.37 to 0.41)	-0.77 (-1.70 to 0.16)	-0.44 (-1.35 to 0.47)
6 months	0.12 (-0.80 to 1.04)	0.10 (-0.79 to 1.00)	0.22 (-0.72 to 1.15)	0.14 (-0.78 to 1.06)
Overall (average)	-1.43 (-2.15 to -0.72)[1]	-0.52 (-1.21 to 0.18)	-1.34 (-2.07 to -0.61)[3]	-0.47 (-1.20 to 0.25)
**Secondary outcomes**				
WOMAC				
2 months	-14.8 (-20.9 to -8.64)[3]	-6.68 (-12.6 to -0.76)[1]	-15.73 (-23.20 to -8.26)[3]	-7.26 (-14.55 to 0.04)
4 months	-6.38 (-12.5 to -0.21)[1]	-6.42 (-12.4 to -0.45)[1]	-7.22 (-14.76 to 0.32)	-6.71 (-14.09 to 0.66)
6 months	-1.42 (-7.68 to 4.84)	-0.78 (-6.82 to 5.27)	-2.40 (-9.95 to 5.15)	-1.48 (-8.87 to 5.90)
Overall	-7.52 (-13.00 to -2.04)[2]	-4.62 (-9.91 to 0.67)	-8.45 (-15.45 to -1.45)[1]	-5.15 (-12.01 to 1.71)
- WOMAC-P	-1.78 (-3.01 to -0.54)[2]	-1.07 (-2.26 to 0.12)	-1.78 (-3.15 to -0.41)[1]	-1.02 (-2.36 to 0.32)
- WOMAC-S	-0.53 (-1.08 to 0.01)	-0.23 (-0.76 to 0.30)	-0.45 (-1.03 to 0.14)	-0.24 (-0.81 to 0.33)
- WOMAC-F	-5.47 (-9.41 to -1.53)[2]	-3.60 (-7.40 to 0.21)	-6.35 (-11.52 to -1.18)[1]	-3.97 (-9.03 to 1.08)
**PSEQ**				
2 months	9.27 (5.10 to 13.4)[3]	6.18 (2.15 to 10.2)[2]	9.14 (3.90 to 14.38)[2]	4.60 (-0.51 to 9.71)
4 months	6.71 (2.51 to 10.9)[2]	5.27 (1.21 to 9.34)[1]	6.66 (1.37 to 11.95)[1]	3.81 (-1.35 to 8.97)
6 months	1.64 (-2.62 to 5.90)	2.88 (-1.24 to 7.00)	2.02 (-3.27 to 7.32)	1.85 (-3.04 to 6.74)
Overall	5.87 (2.30 to 9.45)[2]	4.78 (1.32 to 8.23)[2]	5.94 (1.13 to 10.75)[1]	3.46 (-1.25 to 8.16)
**IPQ**				
2 months	-6.04 (-9.23 to -2.84)[3]	-2.55 (-5.59 to 0.48)	-5.80 (-9.23 to -2.27)[2]	-2.48 (-5.78 to 0.83)
6 months	-0.15 (-3.44 to 3.14)[2]	0.79 (-2.33 to 3.91)	-0.21 (-3.73 to 3.31)	0.64 (-2.75 to 4.04)
Overall	-3.10 (-5.92 to -0.27)[1]	-0.88 (-3.57 to 1.80)	-3.01 (-6.10 to 0.09)	-0.92 (-3.91 to 2.08)
- Consequences	-0.55 (-1.27 to 0.17)	-0.35 (-1.04 to 0.35)	-0.48 (-1.28 to 0.32)	-0.36 (-1.13 to 0.42)
- Timeline	0.07 (-0.74 to 0.60)	0.40 (-0.25 to 1.05)	0.07 (-0.59 to 0.73)	0.30 (-0.34 to 0.94)
- Personal control	0.12 (-0.93 to 0.69)	0.25 (-0.52 to 1.03)	0.22 (-0.63 to 1.06)	0.30 (-0.52 to 1.11)
- Treatment control	2.13 (1.11 to 3.15)[3]	0.98 (0.01 to 1.95)[1]	2.04 (1.05 to 3.04)[3]	0.95 (-0.02 to 1.91)
- Emotional response	-0.63 (-1.43 to 0.16)	-0.43 (-1.19 to 0.33)	-0.58 (-1.53 to 0.38)	-0.17 (-1.10 to 0.76)
**SF-PCS**				

2 months	5.30 (2.38 to 8.21)[3]	4.05 (1.20 to 6.89)[1]	5.84 (2.33 to 9.35)[2]	4.68 (1.23 to 8.12)[2]
4 months	3.07 (0.13 to 6.01)[1]	5.31 (2.41 to 8.21)[3]	3.61 (-0.07 to 7.14)[1]	5.91 (2.41 to 9.41)[2]
6 months	3.04 (0.00 to 6.08)	2.15 (-0.81 to 5.11)	3.63 (0.02 to 7.24)[1]	2.86 (-0.68 to 6.40)
Overall	3.80 (1.33 to 6.27)[2]	3.84 (1.43 to 6.24)[2]	4.36 (1.21 to 7.51)[2]	4.48 (1.39 to 7.58)[2]
**SF-MCS**				
2 months	1.09 (-2.69 to 4.87)	-1.05 (-4.73 to 2.63)	2.42 (-2.09 to 6.92)	0.06 (-4.36 to 4.48)
4 months	2.01 (-1.80 to 5.83)	0.33 (-3.44 to 4.10)	3.44 (-1.10 to 7.99)	-0.49 (-5.00 to 4.03)
6 months	-2.63 (-6.61 to 1.35)	-0.50 (-4.37 to 3.37)	-0.68 (-5.34 to 3.99)	-1.04 (-5.62 to 3.55)
Overall	0.16 (-2.83 to 3.15)	0.29 (-2.60 to 3.19)	1.72 (-2.13 to 5.59)	-0.49 (-4.29 to 3.31)
**EQ-5D-5L**				
2 weeks	0.18 (0.12 to 0.24)[3]	0.06 (0.00 to 0.12)	0.17 (0.08 to 0.26)[3]	0.06 (-0.03 to 0.15)
2 months	0.15 (0.08 to 0.22)[3]	0.07 (0.00 to 0.14)[1]	0.14 (0.05 to 0.23)[2]	0.07 (-0.02 to 0.16)
4 months	0.12 (0.04 to 0.19)[2]	0.10 (0.03 to 0.18)[2]	0.10 (0.01 to 0.19)[1]	0.10 (0.01 to 0.19)[1]
6 months	0.01 (-0.07 to 0.08)	0.00 (-0.07 to 0.08)	0.01 (-0.10 to 0.08)	0.003 (-0.09 to 0.09)
Overall	0.11 (0.06 to 0.17)[3]	0.06 (0.01 to 0.11)[1]	0.10 (0.02 to 0.18)[1]	0.06 (-0.02 to 0.14)
**SPS**				
2 months	4.06 (1.75 to 6.38)[2]	3.40 (1.22 to 5.59)[2]	3.61 (0.80 to 6.43)[1]	3.21 (0.54 to 5.87)[1]
6 months	2.16 (-0.20 to 4.53)	2.45 (0.22 to 4.69)[1]	1.76 (-1.10 to 4.62)	2.31 (-0.39 to 5.01)
Overall	3.11 (1.05 to 5.18)[1]	2.93 (0.98 to 4.88)[1]	2.69 (0.07 to 5.30)[1]	2.76 (0.28 to 5.24)[1]
**Work performance**				
2 months	-1.72 (-2.93 to -0.51)[1]	-1.49 (-2.64 to -0.34)[1]	-1.12 (-2.58 to 0.35)	-1.83 (-3.19 to -0.46)[2]
6 months	-0.84 (-2.08 to 0.39)	-0.47 (-1.64 to 0.71)	-0.21 (-1.69 to 1.27)	-0.70 (-2.09 to 0.69)
Overall	-1.28 (-2.39 to -0.18)[1]	-0.98 (-2.03 to 0.07)	-0.66 (-2.04 to 0.71)	-1.27 (-2.55 to 0.02)
**BMI**				
6 months	-0.22 (-0.91 to 0.46)	0.10 (-0.55 to 0.74)	0.62 (-1.86 to 3.09)	-0.79 (-3.20 to 1.63)
**Perceived change†**				
2 weeks	6.93 (3.39 to 14.2)[3$]	2.28 (1.42 to 3.66)[3]	6.92 (3.40 to 14.10)[3$]	2.25 (1.40 to 3.62)[2]
2 months	6.66 (2.48 to 17.9)[3]	2.63 (1.43 to 4.82)[2]	6.59 (2.46 to 17.64)[3]	2.69 (1.147 to 4.90)[2]
4 months	1.54 (0.32 to 1.32)	1.85 (0.88 to 3.88)	1.52 (0.75 to 3.07)	1.87 (0.89 to 3.90)
6 months	0.91 (0.55 to 2.17)	1.27 (0.62 to 2.58)	1.07 (0.54 to 2.12)	1.27 (0.63 to 2.58)
**Sleep problem†**				
2 months	1.96 (1.28 to 3.03)[2]	1.72 (1.12 to 2.63)[1]	1.66 (1.07 to 2.58)[1]	1.58 (1.01 to 2.44)[1]
4 months	1.27 (0.79 to 2.04)	1.56 (1.06 to 2.27)[1]	1.04 (0.65 to 1.67)	1.49 (1.00 to 2.23)[1]
6 months	1.05 (0.69 to 1.45)	1.10 (0.79 to 1.52)	0.80 (0.54 to 1.20)	1.01 (0.72 to 1.43)
**Rating of overall results of care**				
2 months	2.51 (1.40 to 3.61)[3]	1.43 (0.38 to 2.47)[2]	2.45 (1.36 to 3.54)[3]	1.40 (0.35 to 2.44)[2]
6 months	1.60 (0.50 to 2.71)[2]	0.15 (-0.91 to 1.21)	1.54 (0.45 to 2.63)[2]	0.11 (-0.95 to 1.17)

**Satisfaction with information received**†				
2 months	1.35 (1.10 to 1.64)[2]	1.15 (0.99 to 1.34)	1.33 (1.09 to 1.62)[2]	1.15 (0.98 to 1.34)
6 months	1.19 (0.91 to 1.56)	1.15 (0.94 to 1.40)	1.18 (0.90 to 1.54)	0.87 (0.71 to 1.06)
**Understanding of hip problem**†				
2 months	1.01 (0.87 to 1.17)	1.02 (0.89 to 1.17)	1.02 (0.89 to 1.17)	1.02 (0.89 to 1.17)
6 months	0.98 (0.81 to 1.18)	0.88 (0.76 to 1.03)	0.88 (0.75 to 1.03)	0.88 (0.75 to 1.03)
**Patient still has questions about their hip problem**†				
2 months	1.19 (0.76 to 1.82)	1.16 (0.76 to 1.75)	1.16 (0.75 to 1.77)	1.17 (0.77 to 1.77)
6 months	0.96 (0.65 to 1.43)	1.14 (0.80 to 1.61)	0.95 (0.65 to 1.38)	1.11 (0.79 to 1.56)
**Patient has been kept from their usual activities because of the hip pain**†				
2 months	1.79 (1.15 to 2.70)[2]	1.41 (0.91 to 2.22)	1.66 (1.08 to 2.55)[1]	1.45 (0.66 to 2.26)
6 months	1.15 (0.79 to 1.69)	1.08 (0.76 to 1.54)	1.04 (0.72 to 1.49)	1.03 (0.71 to 1.47)
**Satisfaction with care received**†				
2 months	1.97 (1.33 to 2.93)[2]	1.61 (1.17 to 2.21)[2]	1.93 (1.30 to 2.86)[2]	1.59 (1.16 to 2.19)[2]
6 months	1.72 (1.11 to 2.66)[1]	0.93 (0.69 to 1.25)	1.68 (1.09 to 2.60)[1]	1.14 (0.88 to 1.47)
**Patient would have the same care again if they had the same condition**†				
2 months	2.41 (1.53 to 3.80)[3]	1.45 (1.07 to 1.96)[1]	2.40 (1.52 to 3.80)[3]	1.44 (1.06 to 1.95)[1]
6 months	1.89 (1.22 to 2.92)[2]	1.06 (0.80 to 1.40)	1.87 (1.21 to 2.88)[2]	1.04 (0.79 to 1.38)
**Expectation for pain relief met**†				
2 months	1.52 (1.10 to 2.10)[1]	1.26 (0.95 to 1.67)	1.43 (1.04 to 1.98)[1]	1.24 (0.94 to 1.64)
6 months	1.35 (0.99 to 1.84)	1.29 (0.95 to 1.73)	1.26 (0.92 to 1.73)	1.27 (0.94 to 1.72)

BCT = Best Current Treatment; BCT-US-Triamcinolone-Lidocaine = BCT plus Ultrasound guided injection of triamcinolone and lidocaine; BCT-US-Lidocaine = BCT plus Ultrasound guided injection of lidocaine only.
For numerical outcomes, effects shown are mean differences (summarised by mean scores (SD)). † For binary/categorical outcomes, the Relative Risk (RR) is shown. All effect sizes are presented with their associated 95% Confidence Interval. Adjusted analysis completed by use of linear or generalised mixed models accounting for repeated measures and adjusted for age, gender, baseline pain and (where applicable) the corresponding baseline value.
Outcomes (numerical scales): Western Ontario and McMaster Universities Osteoarthritis Index (WOMAC-T) 0-96 [0=Minimum problems, 96=maximum problems]; WOMAC–Pain subscale (WOMAC-P) 0-20 [0=No pain, 20=Highest pain], WOMAC-Stiffness subscale (WOMAC-S) 0-8 [0=No stiffness, 8=Most stiffness], WOMAC-Function subscale (WOMAC-F) 0-68 [0=No difficulty, 68=Most difficulty]; Pain Self-Efficacy Questionnaire (PSEQ), 0-60 [0=No confidence; 60=Highest confidence]; modified brief Illness Perceptions Questionnaire (IPQ), 0-50 [0=Full understanding, 50=Least understanding]; IPQ-Consequences subscale [0=No affect at all; 10=Severely affects life]; IPQ-Timeline subscale [0=Last very short time; 10=Last forever]; IPQ-Personal control subscale [0=No control; 10=Extreme control]; IPQ-Treatment control subscale [0=Treatment no help; 10=Treatment extremely helpful]; IPQ-Emotional response subscale [0=Not affected emotionally; 10=Extremely affected emotionally]; Short Form-12 Physical Component Scale (SF12-PCS) 0-100 [0=Worst physical health, 100=Best physical health]; Short Form-12 Mental Component Scale (SF12-MCS) 0-100 [0=Worst mental health, 100=best mental health]; EQ5D (utility) -0.59 – 1.00 [-0.59=Worst health utility, 1.00=Best health utility]; Stanford Presenteeism Scale (SPS) 6-30 [6=Minimum ability, 30=Maximum ability]; Work Performance 0-10 numerical integer scale [0=Not affected, 10=Unable to do job]; BMI=Body Mass Index (kg/m^2); Rating of overall results of care: 0-10 numerical integer scale [0=Terrible, 10=Excellent].

[1] 0.01≤P<0.05; [2] 0.001≤P<0.01; [3] P<0.001. $ indicates where RR was derived using completely/much/somewhat better as ‘positive response’ since the number of people who were completely or much better at 2 weeks in the BCT arm was zero and could not be analysed

Supplementary Table 5: Summary of WOMAC subscales (Pain, Stiffness and Function) subscales by treatment arm.

	BCT	BCT-US-Triamcinolone-Lidocaine	BCT-US-Lidocaine	BCT-US-Triamcinolone-Lidocaine v BCT alone	BCT-US-Triamcinolone-Lidocaine v BCT-US-Lidocaine
	mean (SD), n	***mean (SD), n***	***mean (SD), n***	***mean difference* (95% CI) [P value]***	***mean difference* (95% CI) [P value]***
**Pain**					
2 months	10.7 (3.8), 57	7.0 (4.3), 61	8.7 (4.1), 62	-3.61 (-5.02 to -2.20) [P<0.001]	-1.74 (-3.10 to -0.37) [P=0.01]
4 months	9.0 (4.6), 53	7.9 (4.3), 59	9.1 (4.1), 59	-1.25 (-2.67 to 0.18) [P=0.09]	-1.24 (-2.62 to 0.14) [P=0.08]
6 months	9.0 (4.5), 53	8.8 (4.3), 56	9.1 (4.1), 60	-0.47 (-1.91 to 0.97) [P=0.52]	-0.24 (-1.63 to 1.15) [P=0.74]
Overall	9.9 (4.2), 61	8.0 (4.0), 64	9.1 (3.7), 65	-1.78 (-3.01 to -0.54) [P=0.005]	-1.07 (-2.26 to 0.12) [P=0.08]
**Stiffness**					
2 months	4.3 (1.9), 57	3.2 (1.9), 63	3.7 (1.7), 63	-1.24 (-1.87 to -0.62) [P<0.001]	-0.53 (-1.14 to 0.08) [P=0.09]
4 months	3.8 (1.9), 53	3.7 (1.9), 60	3.8 (1.8), 60	-0.25 (-0.89 to 0.39) [P=0.45]	-0.12 (-0.73 to 0.50) [P=0.71]
6 months	3.7 (1.9), 53	3.7 (1.7), 56	3.8 (1.8), 59	-0.11 (-0.75 to 0.54) [P=0.75]	-0.05 (-0.68 to 0.57) [P=0.87]
Overall	3.9 (1.9), 61	3.5 (1.8), 65	3.8 (1.8), 65	-0.53 (-1.08 to 0.01) [P=0.06]	-0.23 (-0.76 to 0.30) [P=0.39]
**Function**					
2 months	35.3 (16.1), 55	23.8 (15.0), 62	29.1 (14.3), 63	-10.4 (-14.8 to 5.95) [P<0.001]	-4.94 (-9.19 to -0.70) [P=0.02]
4 months	30.7 (17.2), 51	26.7 (15.1), 60	31.3 (13.5), 59	-4.81 (-9.23 to -0.37) [P=0.03]	-4.89 (-9.16 to -0.61) [P=0.03]
6 months	30.2 (16.7), 53	28.8 (15.2), 57	31.0 (14.3), 59	-1.25 (-5.74 to 3.25) [P=0.59]	-0.96 (-5.30 to 3.38) [P=0.67]
Overall	32.1 (16.7), 60	26.4 (15.1), 65	30.4 (14.0), 65,	-5.47 (-9.41 to -1.53) [P=0.007]	-3.60 (-7.40 to 0.21) [P=0.06]

BCT = Best Current Treatment; BCT-US-Triamcinolone-Lidocaine = BCT plus Ultrasound guided injection of triamcinolone and lidocaine; BCT-US-Lidocaine = BCT plus Ultrasound guided injection of lidocaine only; n (number of subjects analysed).

Western Ontario and McMaster Universities Osteoarthritis Index (WOMAC-T) 0-96 [0=Minimum problems, 96=maximum problems]; WOMAC–Pain subscale (WOMAC-P) 0-20 [0=No pain, 20=Highest pain], WOMAC-Stiffness subscale (WOMAC-S) 0-8 [0=No stiffness, 8=Most stiffness], WOMAC-Function subscale (WOMAC-F) 0-68 [0=No difficulty, 68=Most difficulty].

* Analysis by longitudinal linear mixed model adjusted for age, gender and baseline pain and WOMAC subscale scores.

Supplementary Table 6: Summary of adverse events.

	BCT	BCT-US-Triamcinolone-Lidocaine	BCT-US-Lidocaine
Total number of non-serious adverse events (related or unrelated)	2	34	34
No. of participants afflicted	2	25	26
Expected related non-serious adverse events			
>Whitening of the skin*	NA	4	2
>Thinning of the skin*	NA	2	2
>Bruising*	NA	15	16
>Pain after injection	NA	1	7
>Flushing	NA	4	0
>Other skin problem eg rash	NA	0	2
>Menorrhagia	NA	1	0
>Infection	NA	1	1
>Anxiety after injection	NA	1	1
Discomfort with exercise	2	0	0
Total events	2	29	31
No. of participants afflicted**	NA	21	24
Non-related non-serious adverse events			
>Constipation	0	1	0
>Headache	0	0	1
>Fall	0	0	1
>Restless leg(s)	0	1	0
>Plantar fascitis	0	1	0
>Neuralgia	0	0	1
>Cough	0	1	0
>Numbness in leg	0	1	0
Total events	0	5	3
No. of participants afflicted	0	4	3
Possibly related Serious adverse events			
Death due to subacute bacterial endocarditis	0	1	0
Non-related Serious adverse events			
Death due to intracerebral haemorrhage following thrombectomy for acute thrombotic stroke	0	1	0
Hospitalisation due to myocardial infarction	0	1	0
Incapacitating flare of pre-existing multiple sclerosis	0	0	1
Hospitalisation for colitis of diverticular origin	1	0	0
Hospitalisation for avascular necrosis of hip, and total hip replacement	1	0	0
Hospitalisation for elective percutaneous angioplasty	0	0	1

BCT = Best Current Treatment; BCT-US-Lidocaine = BCT plus Ultrasound guided injection of lidocaine only; BCT-US-Triamcinolone-Lidocaine = BCT plus Ultrasound guided injection of triamcinolone and lidocaine; NA not applicable

*Specific questions on 2-week and 2-month questionnaire. Recipients of injection were asked to complete a section on side effects and state if any other side effects from injection received.

**For 'No of participants afflicted' in the above table, the estimates below for the CIs were extracted via the Wilson's score method (with continuity correction):-

BCT-US-Triamcinolone-Lidocaine v BCT alone: Risk difference = 0.288 (0.153 to 0.418) → NNH = 3.47 (2.39 to 6.54)

BCT-US-Triamcinolone-Lidocaine v BCT-US-Lidocaine: Risk difference = -0.045 (-0.212 to 0.124) → NNH = -22.2 (-4.72 to 8.06)

Supplementary Table 7: Sensitivity analysis of pain intensity NRS scores (through multiple imputation analysis).

Sensitivity analysis number	Time-point	BCT-US-Triamcinolone-Lidocaine v BCT	BCT-US-Triamcinolone-Lidocaine v BCT-US-Lidocaine
		mean difference (95% CI) [P value]	***mean difference (95% CI) [P value]***
MI - 1	*2 weeks*	-3.13 (-3.96 to -2.31) [P<0.001]	-1.08 (-1.90 to -0.27) [P=0.01]
	2 months	-1.72 (-2.66 to -0.79) [P<0.001]	-0.68 (-1.58 to 0.22) [P=0.14]
	4 months	-0.76 (-1.76 to 0.25) [P=0.14]	-0.39 (-1.34 to 0.55) [P=0.41]
	6 months	0.18 (-0.74 to 1.11) [P=0.70]	0.09 (-0.82 to 0.99) [P=0.85]
	Overall	-1.36 (-2.09 to -0.62) [P<0.001]	-0.52 (-1.24 to 0.20) [P=0.16]
MI - 2	*2 weeks*	-3.18 (-4.00 to -2.36) [P<0.001]	-1.10 (-1.91 to -0.28) [P=0.009]
	2 months	-1.83 (-2.76 to -0.89) [P<0.001]	-0.66 (-1.56 to 0.24) [P=0.15]
	4 months	-0.80 (-1.82 to 0.21) [P=0.12]	-0.33 (-1.29 to 0.62) [P=0.49]
	6 months	0.09 (-0.84 to 1.01) [P=0.85]	0.09 (-0.82 to 0.99) [P=0.85]
	Overall	-1.43 (-2.17 to -0.69) [P<0.001]	-0.50 (-1.22 to 0.22) [P=0.17]
MI - 3	*2 weeks*	-3.09 (-3.91 to -2.27) [P<0.001]	-1.06 (-1.88 to -0.25) [P=0.01]
	2 months	-1.62 (-2.55 to -0.68) [P=0.001]	-0.69 (-1.59 to 0.22) [P=0.14]
	4 months	-0.71 (-1.70 to 0.29) [P=0.16]	-0.45 (-1.39 to 0.49) [P=0.35]
	6 months	0.27 (-0.65 to 1.20) [P=0.47]	0.08 (-0.82 to 0.99) [P=0.86]
	Overall	-1.28 (-2.02 to -0.55) [P=0.001]	-0.53 (-1.25 to 0.19) [P=0.15]
MI - 4	*2 weeks*	-3.24 (-4.07 to -2.40) [P<0.001]	-1.11 (-1.94 to -0.28) [P=0.009]
	2 months	-1.95 (-2.89 to -1.02) [P<0.001]	-0.64 (-1.54 to 0.27) [P=0.17]
	4 months	-0.87 (-1.90 to 0.15) [P=0.09]	-0.26 (-1.23 to 0.71) [P=0.60]
	6 months	-0.06 (-1.00 to 0.88) [P=0.90]	0.08 (-1.00 to 0.85) [P=0.87]
	Overall	-1.53 (-2.28 to -0.79) [P<0.001]	-0.48 (-1.22 to 0.25) [P=0.20]
MI - 5	*2 weeks*	-3.01 (-3.84 to -2.18) [P<0.001]	-1.04 (-1.86 to -0.22) [P=0.01]
	2 months	-1.45 (-2.40 to -0.51) [P=0.003]	-0.69 (-1.61 to 0.23) [P=0.14]
	4 months	-0.65 (-1.63 to 0.34) [P=0.20]	-0.52 (-1.47 to 0.42) [P=0.28]
	6 months	0.39 (-0.54 to 1.33) [P=0.41]	0.08 (-0.84 to 1.01) [P=0.86]
	Overall	-1.18 (-1.92 to -0.44) [P=0.002]	-0.54 (-1.27 to 0.19) [P=0.15]

BCT = Best Current Treatment; BCT-US-Lidocaine = BCT plus Ultrasound guided injection of lidocaine only; BCT-US-Triamcinolone-Lidocaine = BCT plus Ultrasound guided injection of triamcinolone and lidocaine

Sensitivity analysis number: Multiple Imputation (MI) via chained equations [1] MAR analysis; [2-5] NMAR analysis ([2] MAR plus 1-point higher imputation, [3] MAR plus 1-point lower imputation, [4] MAR plus 2.5-points higher imputation, [5] MAR plus 2.5-points lower imputation). Analyses were by linear regression adjusted for age, gender and baseline pain score

Supplementary Table 8: Evaluation of pre-specified exploratory subgroup analyses for the pain intensity score NRS measure.

Baseline variables	BCT	BCT-US-Triamcinolone-Lidocaine	BCT-US-Lidocaine	BCT-US-Triamcinolone-Lidocaine v BCT	BCT-US-Triamcinolone-Lidocaine v BCT-US-Lidocaine
	mean (SD)	***mean (SD)***	***mean (SD)***	***mean difference (95% CI)***	***mean difference (95% CI)***
Pain					
< 5	4.7 (2.5)	3.9 (2.6)	4.1 (2.3)	-0.84	-0.21
≥ 5	6.0 (2.7)	4.3 (2.8)	5.0 (2.6)	(-2.26 to 0.59)	(-1.76 to 1.38)
Duration					
< 6 months	5.6 (3.4)	4.2 (2.7)	4.0 (3.4)	-0.34	-0.85
≥ 6 months	5.6 (2.5)	4.1 (2.7)	4.7 (2.5)	(-2.43 to 1.76)	(-3.49 to 1.79)
IPQ					
< 30	4.6 (2.5)	3.7 (2.7)	4.2 (2.4)	-0.84	-0.21
≥ 30	6.5 (2.5)	4.6 (2.8)	5.1 (2.7)	(-2.26 to 0.59)	(-1.63 to 1.21)
BMI					
< 25	5.5 (2.8)	3.3 (2.3)	5.0 (2.2)	0.54	1.35
≥ 25	5.7 (2.6)	4.3 (2.8)	4.4 (2.6)	(-1.25 to 2.34)	(-0.44 to 3.13)
Received preferred treatment					
No	5.7 (2.6)	3.0 (1.9)	3.6 (2.0)	2.74	-0.50
Yes	3.7 (2.7)	4.2 (2.8)	4.9 (2.5)	(-0.35 to 5.82)	(-3.87 to 2.87)
Presence of obvious synovitis/ effusion*					
No	-	4.2 (3.0)	4.0 (2.5)	-	-1.70
Yes	-	4.2 (2.5)	5.5 (2.4)		(-3.10 to -0.30)†

BCT = Best Current Treatment; BCT-US-Triamcinolone-Lidocaine = BCT plus Ultrasound guided injection of triamcinolone and lidocaine; BCT-US-Lidocaine = BCT plus Ultrasound guided injection of lidocaine only; IPQ = Illness Perceptions Questionnaire; BMI = Body Mass Index.

The between-group estimates are derived from the interaction term for treatment group x baseline variable and where the dependent variable is the numerical pain NRS at follow up and including the baseline covariates of age, gender and baseline pain score.

* Injection subgroups only.

† P=0.017 (magnitude of between-group differences across time were: -0.8 at 2 weeks, -1.9 at 2 months, -1.9 at 4 months, -1.9 at 6 months)
